# Supplementary material for: Characterising Kenyan hospitals’ suitability for medical officer internship training: a secondary data analysis of a cross-sectional study
Source: BMJ Open. 2022 May 6;12(5):e056426. doi: 10.1136/bmjopen-2021-056426 (PMC9083393; doi:10.1136/bmjopen-2021-056426)
Supplement: Supplementary data [file bmjopen-2021-056426supp001.pdf]

Additional file 1. 166 Indicators included for each general domain and speciality domain

Domain	Indicator	1 – considered available	0 – considered unavailable
**Human resources for health (6)**	At least five medical specialists	- 5 or more specialists	- Less than 5 specialists
	At least one surgeon	- 1 or more	- Less than 1
	At least one internist	- 1 or more	- Less than 1
	At least one paediatrician	- 1 or more	- Less than 1
	At least one obstetrician-gynaecologist	- 1 or more	- Less than 1
	At least one anaesthesiologist	- 1 or more	- Less than 1
**Lab test (31)**	HIV rapid test	- Observed, at least one not expired	- Not observed, reported available but not seen<br>- Not observed, never available
	Syphilis rapid test	- Observed, at least one not expired	- Not observed, reported available but not seen<br>- Not observed, never available
	Urine rapid test for pregnancy	- Observed, at least one not expired	- Not observed, reported available but not seen<br>- Not observed, never available
	Urine dipstick 3	- Observed, at least one not expired	- Not observed, reported available but not seen<br>- Not observed, never available
	Haemoglobin A1c rapid test	- Observed, at least one not expired	- Not observed, reported available but not seen<br>- Not observed, never available
	Colorimeter or haemoglobinometer	- Observed, functional/valid	- Reported (not seen), functional/valid<br>- Not available today
	Glucometer	- Observed, functional/valid	- Reported (not seen), functional/valid<br>- Not available today
	Glucometer test strips/discs	- Observed, functional/valid	- Reported (not seen), functional/valid<br>- Not available today
	Any blood chemistry test	- Yes, onsite	- No, never available
	Renal function test	- Yes, observed, functional/valid	- No, not available today
	Other tests for bilirubin	- Yes, onsite	- Yes, offsite (specimen sent)<br>- No, never available
	ABO blood grouping testing	- Yes, onsite	- Yes, offsite (specimen sent)<br>- No, never available
	Rhesus blood grouping testing	- Yes, onsite	- Yes, offsite (specimen sent)<br>- No, never available
	Cross-match testing	- Yes, onsite	- Yes, offsite (specimen sent)<br>- No, not available today
	Onsite PCR for HIV viral load or HIV early-infant diagnosis	- Yes, onsite	- Yes, offsite (specimen sent)<br>- Yes, not fucntional/not valid<br>- No, not available today
	Xpert MTB/RIF rapid diagnostic testing for TB	- Yes, onsite	- Yes, offsite (specimen sent)<br>- No, not available today
	Urine microscopy	- Yes, observed, functional/valid	- No, not available today
	Malaria smears	- Yes, observed, functional/valid	- No, not available today
	Microscopy test CSF body fluid counts	- Yes, onsite	- Yes, offsite (specimen sent)<br>- No, never available
	Cryptococcal antigen testing	- Yes, onsite	- Yes, offsite (specimen sent)<br>- No, never available
	Gram stain testing	- Yes, onsite	- Yes, offsite (specimen sent)<br>- No, never available
	Culture and sensitivity test	- Yes, onsite	- Yes, offsite (specimen sent)<br>- No, never available
	Blood cultures test	- Yes, onsite	- Yes, offsite (specimen sent)<br>- No, never available
	Fine needle aspiration cytology	- Perform in facility	- Refer for test<br>- Not used
	Core needle biopsy of lump specimen	- Perform in facility	- Refer for test<br>- Not used
	Prostate Specific Antigen (PSA) Testing	- Perform in facility	- Refer for test<br>- Not used
	Blood gas measurement	- Yes, onsite	- Yes, offsite (specimen sent)

			- No, never available
	Cardiac marker (CK, Troponin) test	- Yes, onsite	- Yes, offsite (specimen sent)<br>- No, never available
	PAP smear read in facility	- Yes, read in facility	- Yes, read outside facility<br>- No<br>- 0 PAP smears were conducted with results recorded during the past 12 completed months
	VIA/VILLI read in facility	- Yes, read in facility	- Yes, read outside facility<br>- No<br>- 0 VIA/VILLI were conducted with results recorded during the past 12 completed months
	Prepare and examine tissues or samples for cancer	- Yes	- No
**Oxygen and respiratory support (10)**	Surgery oxygen equipment functioning - Oxygen tank/cylinder with attached pressure gauge, pressure regulator	- Yes	- No<br>- Don't know
	Maternity paediatric-sized oxygen delivery apparatus functioning	- Yes	- No
	Outpatient pulse oximeter functioning	- Yes	- No
	Maternity pulse oximeter functioning	- Yes	- No
	Paediatrics/neonatal oxygen functioning	- Yes	- No
	Emergency pulse oximeter functioning	- Yes	- No
	Ventilator available	- Yes, available now	- No
	Chest tubes available	- Observed, at least one not expired	- Not observed, not available today<br>- Not observed, never available
	Adult CPAP equipment available	- Observed, at least one not expired	- Observed, available but expired<br>- Not observed, reported available but not seen<br>- Not observed, not available today<br>- Not observed, never available
	Emergency paediatrics and neonatal CPAP available	- Observed	- Reported, not seen<br>- Not available
**General equipment (8)**	ECG offered	- Yes	- No
	ECG equipment functioning	- Yes equipment available and functioning	- No equipment not available or not functioning
	Ultrasound offered	- Yes	- No
	Ultrasound equipment functioning	- Yes equipment available and functioning	- No equipment not available or not functioning
	X-ray offered	- Yes	- No
	X-ray equipment functioning	- Yes equipment available and functioning	- No equipment not available or not functioning
	CT scan offered	- Yes	- No
	CT scan equipment functioning	- Yes equipment available and functioning	- No equipment not available or not functioning
**24 7 (8)**	Emergency service with structured triage tool 24/7	- 24-hour emergency service	- 10 hours<br>- No structured triage tool
	Medical officer onsite or oncall in facility 24/7	- MO or Emergency practitioner always available, onsite<br>- MO or Emergency practitioner always available, not onsite but oncall in facility	- MO or Emergency practitioner always available, not in facility<br>- Staff not available 24 hours
	Formal triage tool used 24/7	- Yes	- No
	Emergency surgery and anaesthesia 24/7	- 24-hour emergency service	- 0, 2 or 8 hours
	Emergency radiology 24/7	- 24-hour emergency service	- 0, 5, 9, 10, 12 hours
	Emergency diagnostics 24/7	- 24-hour emergency service	- 0, 7, 10, 12 hours
	Emergency pharmacy 24/7	- 24-hour emergency service	- 8, 9, 10, 12, 15 hours<br>- No dedicated pharmacy in facility
	Blood transfusion	- Yes available now	- No
**IPC (9)**	Facility has IPC guideline	- Yes, observed	- Reported not seen<br>- No
	Technical IPC committee	- Yes	- No
	Dedicated (full-time) IPC staff	- Yes	- No

	Guidelines for cleaning	- Yes, observed	- Reported not seen<br>- No
	Step-by-step techniques for specific tasks	- Yes	- No
	Cleaning roster or schedule specifying responsibility	- Yes	- No
	Cleaning beds observed	- Yes	- No
	Cleaning counters/tables observed	- Yes	- No
	Cleaning toilets observed	- Yes	- No
**Quality and safety (24)**	Monthly quality assurance committee meeting	- Monthly	- Quarterly<br>- Biannually
	Documentation of quality assurance information reviewed	- Yes, documentation observed	- Yes, documentation reported not seen
	Ongoing quality improvement using Plan-Do-Check-Study-Act approach	- Yes, service-specific<br>- Yes, across facility	- No
	Multi-disciplinary quality improvement team	- Yes	- No
	Departmental specific work improvement teams	- Yes	- No
	Documentation of improvement achieved as a result of quality improvement process	- Yes, documentation observed	- Yes, documentation reported not seen<br>- No
	Kenya Quality Model for Health completed in 2017 or 2018	- 2017<br>- 2018	- 2014<br>- 2016<br>- Don't know
	Formal case reviews carried out at least monthly	- At least weekly<br>- At least monthly	- At least quarterly<br>- No specific timing
	Death reviews results recorded	- Yes	- No
	Guidelines of events that are considered adverse and required to be reported	- Yes, documentation observed	- Yes, documentation reported not seen<br>- No
	Notes or reports that show evidence of review and plan of action for the reports about adverse events	- Yes, documentation observed	- Yes, documentation reported not seen<br>- No
	Written guidelines that define nosocomial infections and the process for reporting	- Yes, observed	- Reported not seen<br>- No
	Report or record shows nosocomial infections reported over the past 6 months	- Yes, observed	- Reported not seen<br>- No
	Written policies and procedures for identifying and managing drug-use problems	- Yes, observed	- Reported not seen<br>- No
	Surgery related mortality and morbidity review meetings at least monthly	- At least weekly<br>- At least monthly	- At least quarterly<br>- No specific timing<br>- None conducted
	Surgery related mortality and morbidity review meeting results recorded	- Yes, observed	- Reported not seen<br>- No
	Written guidelines or instructions for reporting on adverse events related to surgery	- Yes, observed	- Reported not seen<br>- No<br>- Don't know
	Notes or reports that show evidence of review and plan of action for the surgery reports	- Yes, documentation observed	- Yes, documentation reported not seen<br>- No
	Guidelines of postoperative infection definition	- Yes, documentation observed	- Yes, documentation reported not seen<br>- No
	Notes or reports that show evidence of review and plan of action for the reports about postoperative infection	- Yes, documentation observed	- Yes, documentation reported not seen<br>- No
	Indicator on deaths prior to discharge among patients who had a procedure in a surgical theater monitored	- Yes	- No
	Indicator on deaths prior to discharge among < 15 year old patients who had a procedure in a surgical theater monitored	- Yes	- No
	Indicator on post-operative surgical wound infection numbers monitored	- Yes	- No
	Facility monitor unplanned and unexpected hospital readmissions for any conditions	- Yes	- No
**Surgery equipment and medication (8)**	Basic operating table functioning	- Yes	- No
	Overhead operating light functioning	- Yes	- No
	Cardiac monitor functioning	- Yes	- No

			- Don't know
	EKG electrodes functioning	- Yes	- No<br>- Don't know
	Thermometer functioning	- Yes	- No<br>- Don't know
	Blood pressure apparatus functioning	- Yes	- No
	Suction apparatus functioning	- Yes	- No
	Anaesthesia machine available	- Yes available now	- No
**Surgery service (12)**	Wound debridement service available	- Yes, outpatient<br>- Yes, inpatient<br>- Yes both out and inpatient	- No
	Acute burn management service available	- Yes, outpatient<br>- Yes, inpatient<br>- Yes both out and inpatient	- No
	Closed repair of fracture service available	- Yes, outpatient<br>- Yes, inpatient<br>- Yes both out and inpatient	- No
	Closed reduction of dislocated joint service available	- Yes, outpatient<br>- Yes, inpatient<br>- Yes both out and inpatient	- No
	Cricothyroidotomy service available	- Yes, outpatient<br>- Yes, inpatient<br>- Yes both out and inpatient	- No
	Male circumcision service available	- Yes, outpatient<br>- Yes, inpatient<br>- Yes both out and inpatient	- No
	Hydrocele reduction service available	- Yes, outpatient<br>- Yes, inpatient<br>- Yes both out and inpatient	- No
	Biopsy of lymph node or mass service available	- Yes, outpatient<br>- Yes, inpatient<br>- Yes both out and inpatient	- No
	Appendectomy service available	- Yes, outpatient<br>- Yes, inpatient<br>- Yes both out and inpatient	- No
	Hernia repair service available	- Yes, outpatient<br>- Yes, inpatient<br>- Yes both out and inpatient	- No
	Open reduction and fixation service available	- Yes, outpatient<br>- Yes, inpatient<br>- Yes both out and inpatient	- No
	Any procedures using laparotomy service available	- Yes, outpatient<br>- Yes, inpatient<br>- Yes both out and inpatient	- No
**Internal medicine equipment and medication (3)**	Defibrillator available	- Yes, available now	- No
	Renal dialysis/haemodialysis machine available	- Yes, available now	- Yes, not available now<br>- No
	Lumbar puncture kit available	- Observed, at least one not expired	- Observed, available but expired<br>- Not observed, reported available but not seen<br>- Not observed, not available today<br>- Not observed, never available
**Internal medicine service (10)**	ART prescription and follow-up for U5 children service available	- Yes, ART prescription and clinical follow-up	- Yes, ART prescription, no clinical follow-up<br>- Yes, clinical follow-up, no ART prescription
	HIV/AIDS care and support service available	- Yes	- No
	Diagnose and/or manage diabetes available	- Yes	- No
	Diagnose and/or manage acute myocardial infraction available	- Diagnose, treat and patient follow-up	- Patient follow-up only<br>- No service/refer suspect case
	Diagnose and/or manage chronic respiratory disease available	- Yes	- No
	Diagnose and/or manage chronic kidney disease available	- Yes	- No
	Screening, diagnosis and/or treatment services for breast cancer available	- Yes	- No

	Screening, diagnosis and/or treatment services for colorectal cancer available	- Yes	- No
	Screening, diagnosis and/or treatment services for prostate cancer available	- Yes	- No
	Palliative care service available	- Yes	- No
**Paediatrics equipment and medication (8)**	Phototherapy machine available	- Yes, available now	- No
	Infant incubator available at outpatient	- Yes, available now	- Yes, not available now<br>- No
	Bed or location for KMC caregiver overnight	- Yes, observed	- Reported, not seen<br>- No
	Exchange transfusion blood available today	- Yes	- No
	Incubator available today	- Yes	- No
	Radiant warmer available today	- Yes	- No
	Artificial ventilation available today	- Yes	- No
	Device for intraosseous access available	- Observed, at least one not expired	- Observed, available but expired<br>- Not observed, reported available but not seen<br>- Not observed, not available today<br>- Not observed, never available
**Paediatrics service (3)**	KMC for premature or LBW used	- Yes	- No
	Newborn sepsis service available	- Yes	- No
	Newborn sepsis full antibiotics regimen and follow-up always available	- Yes always	- Yes sometimes<br>- Never
**OBGYN equipment and medication (10)**	Blood pressure apparatus functioning	- Yes	- No
	Infant scale (with 100 gram gradation) functioning	- Yes	- Don't know
	Resuscitation table (with heat source) functioning	- Yes	- No
	Self-inflating bag and mask for resuscitation functioning	- Yes	- No
	Newborn bag and mask size 0 for resuscitation of pre-term babies functioning	- Yes	- No
	Newborn bag and mask size 1 for resuscitation of term babies functioning	- Yes	- No
	Magnesium sulphate injection available	- Observed, at least one not expired	- Not observed, not available today
	Dexamethasone injection available	- Observed, at least one not expired	- Not observed, not available today<br>- Not observed, never available
	Misoprostol tablet 200 mcg available	- Observed, at least one not expired	- Not observed, not available today<br>- Not observed, never available
	Oxytocin injection available	- Observed, at least one not expired	- Not observed, not available today<br>- Not observed, never available
**OBGYN service (10)**	Active management of third stage labour (AMTSL) service available	- Yes	- No
	Monitoring and management of labor using a partograph service available	- Yes	- No
	Hygienic cord care service available	- Yes	- No
	Parenteral administration of antibiotics carried out	- Yes	- No
	Parenteral administration of oxytocic for treatment of postpartum haemorrhage carried out	- Yes	- No
	Assisted vaginal delivery using manual vacuum extraction (MVE) or forceps carried out	- Yes	- No, service not offered<br>- No, trained staff but no cases
	Manual removal of placenta carried out	- Yes	- No
	Removal of retained products of conception using D&C or manual vacuum aspiration carried out	- Yes	- No, service not offered
	PMTCT available	- Yes	- No
	Treatment of pre-invasive cervical cancer available	- Yes	- No
**Mental health / neurology (6)**	Any mental/neurological services offered	- Yes, inpatient only	- Not offered
	Mental health inpatient ward available	- Yes, inpatient only	- Not offered

	Neurological health inpatient ward available	- Yes, inpatient only	- Not offered
	Depression diagnosis and follow-up available	- Diagnoses and provides patient follow-up	- Diagnoses only<br>- Provides patient follow-up only<br>- No
	Psychosis diagnosis and follow-up available	- Diagnoses and provides patient follow-up	- Diagnoses only<br>- Provides patient follow-up only<br>- No
	Epilepsy diagnosis and follow-up available	- Diagnoses and provides patient follow-up	- Diagnoses only<br>- Provides patient follow-up only<br>- No
