# Supplementary material for: Characterising Kenyan hospitals’ suitability for medical officer internship training: a secondary data analysis of a cross-sectional study
Source: BMJ Open. 2022 May 6;12(5):e056426. doi: 10.1136/bmjopen-2021-056426 (PMC9083393; doi:10.1136/bmjopen-2021-056426)
Supplement: Supplementary data [file bmjopen-2021-056426supp002.pdf]

Additional file 2. Comparing internship hospitals sampled in KHHFA and others

	61 KHHFA hospitals	12 other hospitals
Level		
- Level 4	44	11
- Level 5	15	1
- Level 6	2	0
Ownership		
- MoH	54	0
- Private	2	4
- FBO	5	8
Bed number		
- Mean (SD)	262.9 (213.4)	171.2 (71.0)
- Median (IQR)	200 (156, 276)	180 (106, 221)
Total delivery		
- Mean	4585.1 (2747.0)	1349.4 (945.7)
- Median (IQR)	3980.7 (2972.3, 5502.7)	1150.4 (649.7, 1683.5)
Live births		
- Mean	4466.9 (2691.9)	1321.3 (927.8)
- Median (IQR)	3814.3 (2844, 5384.3)	1138.4 (626.5, 1630)
C-section		
- Mean	1190.3 (917.6)	536.1 (270.7)
- Median (IQR)	987.3 (734.3, 1319.3)	536.6 (289, 708.5)
