# Supplementary material for: Characterising Kenyan hospitals’ suitability for medical officer internship training: a secondary data analysis of a cross-sectional study
Source: BMJ Open. 2022 May 6;12(5):e056426. doi: 10.1136/bmjopen-2021-056426 (PMC9083393; doi:10.1136/bmjopen-2021-056426)
Supplement: Supplementary data [file bmjopen-2021-056426supp003.pdf]

Additional file 3. All indicator heatmap by hospital bed number

	Level 4 small hospitals (n=23)	Level 4 large hospitals (n=23)	Level 5 & 6 hospitals (n=15)	Total (n=61)
**Human resources for health**				
At least five medical specialists	39%	44%	67%	48%
At least one surgeon	96%	96%	100%	97%
At least one internist	48%	57%	73%	57%
At least one paediatrician	70%	91%	100%	85%
At least one obstetrician-gynaecologist	87%	91%	100%	92%
At least one anaesthesiologist	44%	26%	93%	49%
**Lab tests**				
Rapid test				
HIV rapid test	87%	91%	87%	89%
Syphilis rapid test	96%	91%	100%	95%
Urine rapid test for pregnancy	100%	91%	100%	97%
Urine dipstick 3	35%	44%	53%	43%
Haemoglobin A1c rapid test	57%	57%	87%	64%
Colorimeter or haemoglobinometer	70%	61%	67%	66%
Glucometer	96%	96%	93%	95%
Glucometer test strips/discs	96%	100%	93%	97%
Basic lab test				
Any blood chemistry test	83%	78%	93%	84%
Renal function test	70%	74%	93%	77%
Other tests for bilirubin	65%	70%	67%	67%
ABO blood grouping testing	83%	74%	80%	79%
Rhesus blood grouping testing	83%	74%	87%	80%
Cross-match testing	57%	70%	80%	67%
Infectious disease test				
Onsite PCR for HIV viral load or HIV early-infant diagnosis	0%	4%	27%	8%
Xpert MTB/RIF rapid diagnostic testing for TB	61%	83%	87%	75%
Urine microscopy	91%	83%	93%	89%
Malaria smears	91%	83%	93%	89%
Microscopy test CSF body fluid counts	83%	78%	93%	84%
Cryptococcal antigen testing	74%	78%	80%	77%
Gram stain testing	91%	78%	93%	87%
Culture and sensitivity test	57%	74%	87%	71%
Blood cultures test	17%	22%	80%	34%
Advanced lab test				
Fine needle aspiration cytology	22%	44%	67%	41%
Core needle biopsy of lump specimen	35%	44%	67%	46%
Prostate Specific Antigen (PSA) Testing	48%	61%	80%	61%
Blood gas measurement	9%	13%	60%	23%
Cardiac marker (CK, Troponin) test	9%	30%	53%	28%
PAP smear read in facility	17%	13%	33%	20%
VIA/VILLI read in facility	96%	78%	73%	84%
Prepare and examine tissues or samples for cancer	0%	4%	53%	15%

**Oxygen and respiratory support**				
Surgery oxygen equipment functioning - Oxygen tank/cylinder with attached pressure gauge, pressure regulator	87%	83%	73%	82%
Maternity paediatric-sized oxygen delivery apparatus functioning	87%	74%	93%	84%
Outpatient pulse oximeter functioning	39%	48%	60%	48%
Maternity pulse oximeter functioning	35%	52%	67%	49%
Paediatrics/neonatal oxygen functioning	100%	100%	100%	100%
Emergency pulse oximeter functioning	65%	65%	100%	74%
Ventilator available	52%	52%	87%	61%
Chest tubes available	30%	61%	80%	54%
Adult CPAP equipment available	13%	30%	40%	26%
Emergency paediatrics and neonatal CPAP available	17%	26%	20%	21%
**Equipment**				
ECG offered	39%	35%	93%	51%
ECG equipment functioning	26%	35%	87%	44%
Ultrasound offered	83%	91%	100%	90%
Ultrasound equipment functioning	83%	91%	100%	90%
X-ray offered	87%	87%	100%	90%
X-ray equipment functioning	87%	83%	100%	89%
CT scan offered	26%	22%	80%	38%
CT scan equipment functioning	26%	17%	73%	34%
**24 7 availability**				
Emergency service 24/7	26%	13%	47%	26%
Medical officer onsite or oncall in facility 24/7	78%	70%	93%	79%
Formal triage tool used 24/7	26%	17%	47%	28%
Emergency surgery and anaesthesia 24/7	17%	13%	40%	21%
Emergency radiology 24/7	91%	87%	100%	92%
Emergency diagnostics 24/7	96%	87%	93%	92%
Emergency pharmacy 24/7	87%	83%	93%	87%
Blood transfusion	91%	83%	100%	90%
**Infection prevention and control**				
Facility has IPC guideline	87%	70%	87%	80%
Technical IPC committee	96%	100%	93%	97%
Dedicated (full-time) IPC staff	57%	52%	73%	59%
Guidelines for cleaning	48%	57%	33%	48%
Step-by-step techniques for specific tasks	44%	57%	60%	53%
Cleaning roster or schedule specifying responsibility	48%	61%	73%	59%
Cleaning beds observed	44%	61%	67%	56%
Cleaning counters/tables observed	48%	61%	60%	56%
Cleaning toilets observed	48%	70%	67%	61%
**Quality and safety**				
Monthly quality assurance committee meeting	26%	35%	53%	36%
Documentation of quality assurance information reviewed	52%	44%	67%	53%
Ongoing quality improvement using Plan-Do-Check-Study-Act approach	64%	67%	67%	66%
Multi-disciplinary quality improvement team	65%	61%	80%	67%
Departmental specific work improvement teams	65%	61%	80%	67%
Documentation of improvement achieved as a result of quality improvement process	48%	52%	33%	46%

Kenya Quality Model for Health completed in 2017 or 2018	48%	44%	60%	49%
Formal case reviews carried out at least monthly	57%	57%	67%	59%
Death reviews results recorded	91%	91%	100%	93%
Guidelines of events that are considered adverse and required to be reported	22%	35%	33%	30%
Notes or reports that show evidence of review and plan of action for the reports about adverse events	22%	39%	27%	30%
Written guidelines that define nosocomial infections and the process for reporting	9%	9%	40%	16%
Report or record shows nosocomial infections reported over the past 6 months	9%	4%	33%	13%
Written policies and procedures for identifying and managing drug-use problems	70%	65%	67%	67%
Surgery related mortality and morbidity review meetings at least monthly	44%	39%	60%	46%
Surgery related mortality and morbidity review meeting results recorded	44%	57%	53%	51%
Written guidelines or instructions for reporting on adverse events related to surgery	9%	26%	33%	21%
Notes or reports that show evidence of review and plan of action for the surgery reports	9%	17%	27%	16%
Guidelines of postoperative infection definition	17%	26%	33%	25%
Notes or reports that show evidence of review and plan of action for the reports about postoperative infection	13%	17%	13%	15%
Indicator on deaths prior to discharge among patients who had a procedure in a surgical theater monitored	9%	22%	53%	25%
Indicator on deaths prior to discharge among < 15 year old patients who had a procedure in a surgical theater monitored	9%	17%	47%	21%
Indicator on post-operative surgical wound infection numbers monitored	9%	17%	40%	20%
Facility monitor unplanned and unexpected hospital readmissions for any conditions	30%	26%	40%	31%
**Surgery**				
Surgery equipment & medication				
Basic operating table functioning	96%	96%	100%	97%
Overhead operating light functioning	96%	96%	100%	97%
Cardiac monitor functioning	87%	83%	100%	89%
EKG electrodes functioning	83%	70%	100%	82%
Thermometer functioning	96%	87%	93%	92%
Blood pressure apparatus functioning	70%	91%	93%	84%
Suction apparatus functioning	100%	96%	100%	98%
Anaesthesia machine available	83%	74%	93%	82%
Surgery service				
Wound debridement service available	100%	100%	100%	100%
Acute burn management service available	96%	96%	100%	97%
Closed repair of fracture service available	96%	91%	100%	95%
Closed reduction of dislocated joint service available	96%	91%	100%	95%
Cricothyroidotomy service available	78%	57%	80%	71%
Male circumcision service available	100%	96%	93%	97%
Hydrocele reduction service available	100%	91%	93%	95%
Biopsy of lymph node or mass service available	100%	100%	100%	100%
Appendectomy service available	100%	96%	100%	98%
Hernia repair service available	100%	96%	100%	98%
Open reduction and fixation service available	100%	83%	100%	93%
Any procedures using laparotomy service available	100%	96%	100%	98%
**Internal medicine**				

Internal medicine equipment & medication				
Defibrillator available	57%	39%	73%	54%
Renal dialysis/haemodialysis machine available	44%	65%	93%	64%
Lumbar puncture kit available	22%	26%	80%	38%
Internal medicine service				
ART prescription and follow-up for U5 children service available	96%	96%	93%	95%
HIV/AIDS care and support service available	100%	96%	100%	98%
Diagnose and/or manage diabetes available	100%	100%	100%	100%
Diagnose and/or manage acute myocardial infraction available	83%	74%	93%	82%
Diagnose and/or manage chronic respiratory disease available	100%	100%	100%	100%
Diagnose and/or manage chronic kidney disease available	83%	91%	100%	90%
Screening, diagnosis and/or treatment services for breast cancer available	78%	87%	93%	85%
Screening, diagnosis and/or treatment services for colorectal cancer available	35%	30%	60%	39%
Screening, diagnosis and/or treatment services for prostate cancer available	61%	83%	87%	75%
Palliative care service available	48%	65%	100%	67%
**Paediatrics**				
Paediatrics equipment & medication				
Phototherapy machine available	52%	52%	67%	56%
Infant incubator available	74%	78%	87%	79%
Bed or location for KMC caregiver overnight	61%	70%	80%	69%
Exchange transfusion blood available today	26%	17%	60%	31%
Incubator available today	96%	100%	100%	98%
Radiant warmer available today	91%	96%	93%	93%
Artificial ventilation available today	48%	57%	73%	57%
Device for intraosseous access available	17%	30%	53%	31%
Paediatrics service				
KMC for premature or LBW used	83%	87%	93%	87%
Newborn sepsis service available	100%	96%	100%	98%
Newborn sepsis full antibiotics regimen and follow-up always available	91%	87%	87%	89%
**OBGYN**				
OBGYN equipment & medication				
Blood pressure apparatus functioning	91%	100%	100%	97%
Infant scale (with 100 gram gradation) functioning	87%	96%	100%	93%
Resuscitation table (with heat source) functioning	91%	100%	93%	95%
Self-inflating bag and mask for resuscitation functioning	87%	91%	80%	87%
Newborn bag and mask size 0 for resuscitation of pre-term babies functioning	91%	91%	87%	90%
Newborn bag and mask size 1 for resuscitation of term babies functioning	96%	96%	93%	95%
Magnesium sulphate injection available	96%	96%	100%	97%
Dexamethasone injection available	91%	91%	73%	87%
Misoprostol tablet 200 mcg available	74%	70%	73%	72%
Oxytocin injection available	100%	96%	100%	98%
OBGYN service				
Active management of third stage labour (AMTSL) service available	100%	100%	100%	100%
Monitoring and management of labor using a partograph service available	96%	96%	100%	97%
Hygienic cord care service available	100%	91%	93%	95%

Parenteral administration of antibiotics carried out	100%	100%	100%	100%
Parenteral administration of oxytocic for treatment of postpartum haemorrhage carried out	100%	100%	100%	100%
Assisted vaginal delivery using manual vacuum extraction (MVE) or forceps carried out	57%	74%	73%	67%
Manual removal of placenta carried out	100%	100%	100%	100%
Removal of retained products of conception using D&C or manual vacuum aspiration carried out	83%	83%	100%	87%
PMTCT available	100%	100%	100%	100%
Treatment of pre-invasive cervical cancer available	44%	48%	53%	48%
**Mental health / neurology**				
Any mental/neurological services offered	65%	96%	93%	84%
Mental health inpatient ward available	26%	48%	80%	48%
Neurological health inpatient ward available	13%	22%	67%	30%
Depression diagnosis and follow-up available	48%	87%	87%	72%
Psychosis diagnosis and follow-up available	52%	91%	87%	75%
Epilepsy diagnosis and follow-up available	57%	91%	87%	77%
