# Supplementary material for: Characterising Kenyan hospitals’ suitability for medical officer internship training: a secondary data analysis of a cross-sectional study
Source: BMJ Open. 2022 May 6;12(5):e056426. doi: 10.1136/bmjopen-2021-056426 (PMC9083393; doi:10.1136/bmjopen-2021-056426)
Supplement: Author's manuscript [file bmjopen-2021-056426.draft_revisions.pdf]

BMJ Open

BMJ Open is committed to open peer review. As part of this commitment we make the peer review history of every article we publish publicly available.

When an article is published we post the peer reviewers' comments and the authors' responses online. We also post the versions of the paper that were used during peer review. These are the versions that the peer review comments apply to.

The versions of the paper that follow are the versions that were submitted during the peer review process. They are not the versions of record or the final published versions. They should not be cited or distributed as the published version of this manuscript.

BMJ Open is an open access journal and the full, final, typeset and author-corrected version of record of the manuscript is available on our site with no access controls, subscription charges or pay-per-view fees (http://bmjopen.bmj.com).

If you have any questions on BMJ Open's open peer review process please email info.bmjopen@bmj.com

BMJ Open

Characterising hospitals' suitability for medical officer internship training in Kenya: analysis of the Kenya Harmonized Health Facility Assessment

Journal:	*BMJ Open*
Manuscript ID	bmjopen-2021-056426
Article Type:	Original research
Date Submitted by the Author:	13-Aug-2021
Complete List of Authors:	Zhao, Yingxi; University of Oxford, Nuffield Department of Medicine<br>Osano, Boniface; Kenya Paediatric Research Consortium (KEPRECON)<br>Were, Fred; Kenya Paediatric Research Consortium (KEPRECON)<br>Kiarie, Helen; Ministry of Health<br>Nicodemo, Catia; University of Oxford, CHSEO<br>Gathara, David; Kenya Medical Research Institute, Wellcome Trust Research Program<br>English, Mike; University of Oxford, Nuffield Department of Medicine and Department of Paediatrics; KEMRI-Wellcome Trust Research Programme Nairobi,
Keywords:	Organisation of health services < HEALTH SERVICES ADMINISTRATION & MANAGEMENT, Quality in health care < HEALTH SERVICES ADMINISTRATION & MANAGEMENT, Health & safety < HEALTH SERVICES ADMINISTRATION & MANAGEMENT, MEDICAL EDUCATION & TRAINING

SCHOLARONE™
Manuscripts

I, the Submitting Author has the right to grant and does grant on behalf of all authors of the Work (as defined in the below author licence), an exclusive licence and/or a non-exclusive licence for contributions from authors who are: i) UK Crown employees; ii) where BMJ has agreed a CC-BY licence shall apply, and/or iii) in accordance with the terms applicable for US Federal Government officers or employees acting as part of their official duties; on a worldwide, perpetual, irrevocable, royalty-free basis to BMJ Publishing Group Ltd ("BMJ") its licensees and where the relevant Journal is co-owned by BMJ to the co-owners of the Journal, to publish the Work in this journal and any other BMJ products and to exploit all rights, as set out in our licence.

The Submitting Author accepts and understands that any supply made under these terms is made by BMJ to the Submitting Author unless you are acting as an employee on behalf of your employer or a postgraduate student of an affiliated institution which is paying any applicable article publishing charge ("APC") for Open Access articles. Where the Submitting Author wishes to make the Work available on an Open Access basis (and intends to pay the relevant APC), the terms of reuse of such Open Access shall be governed by a Creative Commons licence – details of these licences and which Creative Commons licence will apply to this Work are set out in our licence referred to above.

Other than as permitted in any relevant BMJ Author's Self Archiving Policies, I confirm this Work has not been accepted for publication elsewhere, is not being considered for publication elsewhere and does not duplicate material already published. I confirm all authors consent to publication of this Work and authorise the granting of this licence.

For peer review only

Characterising hospitals' suitability for medical officer internship training in Kenya: analysis of the Kenya Harmonized Health Facility Assessment

Author: Yingxi Zhao[1], Boniface Osano[2,3], Fred Were[2,3], Helen Kiarie[4], Catia Nicodemo[5], David Gathara[6,7], Mike English[1,6]

Affiliation:

1. Oxford Centre for Global Health Research, Nuffield Department of Medicine, University of Oxford, Oxford, UK
2. University of Nairobi Department of Paediatrics and Child Health, Nairobi, Kenya
3. Kenya Paediatric Research Consortium (KEPRECON), Nairobi, Kenya
4. Division of Monitoring & Evaluation, Ministry of Health, Nairobi, Kenya
5. Nuffield Department of Primary Care Health Sciences, University of Oxford, Oxford, UK
6. KEMRI-Wellcome Trust Research Programme, Nairobi, Kenya
7. MARCH Centre, London School of Hygiene and Tropical Medicine, London, UK

For peer review only

For peer review only - http://bmjopen.bmj.com/site/about/guidelines.xhtml

Corresponding: Yingxi Zhao

+44 (0) 7491422427

yingxi.zhao@ndm.ox.ac.uk

Oxford Centre for Global Health Research

Nuffield Department of Medicine

University of Oxford

S Parks Rd, Oxford OX1 3SY, UK

Version: 3.4 Updated Aug 13th, 2021

Word count: 3,931

For peer review only

Abstract

Objective: To characterise the quality, readiness and capacity of Kenya internship hospitals to understand whether they are suitable to provide internship training for medical doctors.

Design: A secondary data analysis of a cross-sectional health facility assessment.

Setting and population: We used secondary data from Kenya Harmonized Health Facility Assessment (KHFA) 2018, which sampled 61 out of all 74 hospitals that provide internship training for medical doctors in Kenya.

Outcome measures: Comparing against the minimum requirement outlined in the national guidelines for medical officer interns, we filtered and identified 166 indicators from the KHFA survey questionnaire and grouped them into 12 domains. An overall readiness and capacity index was calculated as the mean of 12 domain-specific scores for each facility.

Results: The average overall capacity and readiness index is 69% (95%CI 66%-72%) for all internship training centres. Hospitals have moderate capacity and readiness (over 60%) for most of the general domains, though there is huge variation between hospitals and only 29 out of 61 hospitals have five or more specialists assigned, employed, seconded or part-time - as required by the national guideline. Quality and safety score was low across all hospitals with an average score of 40%. As for major specialties, all hospitals have good readiness and capacity for surgery and obstetrics-gynaecology, while mental health was poorest in comparison. Level 5 & 6 facilities have higher capacity scores in all domains when compared with Level 4 hospitals.

Conclusion: Major gaps exist in staffing, equipment and service availability of Kenya internship hospitals. Level 4 hospitals are more likely to have a lower readiness and capacity index, leading to low quality of care, and should be reviewed and improved to provide

For peer review only

appropriate and well-resourced training for interns and to utilise appropriate resources to avoid improvising.

Keywords

Internship, hospital, capacity, service availability, human resources for health, quality and safety

Strengths and limitations of this study:

- Using data collected from a national health facility assessment, our study is able to characterise the readiness and capacity of 61 out of 74 Kenya internship hospitals and contrast findings across levels of hospitals and with the minimum requirements outlined in the national guidelines for medical officer internship training.
- Our analyses have clear implications for Kenya policy-makers to improve the conditions of internship hospitals. More widely, our findings point to the need to carefully consider the potential consequences of rapidly expanding medical training and of appropriate planning and financing for new internship centres, especially in rural areas.
- Our analysis was only limited to the 61 internship training hospitals sampled by KHFA, mostly public hospitals; and KHFA data was collected n 2018 prior to Covid-19 therefore it is possible that the readiness and capacity of Kenyan internship training centres has improved since 2018.
- To assess the readiness and capacity we selected 166 signal indicators from the 3000 questions from KHFA survey, however the indicators selected focused only on the

For peer review only - http://bmjopen.bmj.com/site/about/guidelines.xhtml

80 structural and organizational features of internship training hospitals, and the
81 selection process could be somewhat subjective.

For peer review only

For peer review only - http://bmjopen.bmj.com/site/about/guidelines.xhtml

Background

In Kenya as in many countries, doctors are first trained for 5 or 6 years in medical school (typically 2 or 3 years pre-clinical, 3 years clinical). This is followed by a mandatory one-year internship prior to receiving a license to practice from the national regulatory board, i.e. the Kenya Medical Practitioners and Dentists Council, overseen by the National Ministry of Health (1). The Kenyan medical internship included supervised rotation in 4 major departments (surgery, internal medicine, paediatrics and child health, and obstetrics and gynaecology [OBGYN]) but since 2020 mental health and community health practice became added requirements. The competencies medical interns are expected to develop are outlined in national guidelines and log books (2). After successfully completing internship and subsequent licensure individuals can practice medicine unsupervised as general medical officers.

Medical internships are undertaken in specific hospitals approved for this purpose after evaluation by the Kenya Medical Practitioners and Dentists Council, and all hospitals should meet and maintain their minimum requirements. These sites are an essential component of the health and education system producing a country's future physicians (3). Ensuring internship training hospitals are well-staffed and equipped is important so that medical graduates can be supervised and supported to consolidate their knowledge and skills, including of common clinical procedures, and become competent medical doctors (4), as well as improving quality of care and patient safety, as interns are at the frontline of patient management especially in LMICs (5).

For peer review only - http://bmjopen.bmj.com/site/about/guidelines.xhtml

As with other African countries, medical schools in Kenya are expanding to meet human resources for health gaps. The number of Kenyan medical schools has expanded dramatically from 2 in 2004 to 12 in 2018 (6), meanwhile the number of medical doctors graduating almost doubled from 287 in 2006 to 573 in 2018 (7,8). Correspondingly, the number of internship training centres recognised by the Ministry of Health and regulators increased from 6 to 74 over the past 15 years and includes private or mission/faith-based hospitals. (9,10). Many new training centres that were formerly district hospitals but are now county hospitals in Kenya (at Level 4 of its health system) became recognised as internship centres alongside more established regional and national hospitals (at Level 5 and 6 respectively). However, Level 4 hospitals may have shortages of staff, limited diagnostic facilities, and frequent stockout of medications and diagnostics reagents, and are “not organized for training purposes” according to a qualitative study in Kenya (9).

Previous studies conducted in Kenya between 2012 and 2014 highlighted poor working conditions and experiences for interns at district hospitals, however they were limited in scale (all including less than 10 hospitals) and did not compare hospitals at different levels and sizes (9,11,12). Using data collected from the Kenya Harmonized Health Facility Assessment 2018 (KHFA), we address the question ‘what capacity do facilities offering internship training have?’ To do this we characterise the readiness and capacity of 61 internship hospitals contrasting findings across levels of hospitals and with the minimum requirements outlined in the national guidelines for medical officer internship training (2), with a specific focus on the ‘structural’ component of the Donabedian quality of care framework.

For peer review only

For peer review only - http://bmjopen.bmj.com/site/about/guidelines.xhtml

Methods

Data sources

This is a secondary data analysis using data from the 2018 Kenya Harmonized Health Facility Assessment (KHFA). Details of the KHFA and its methodology are publicly available (13). KHFA used the Kenya Health Master Facility List (MFL) as the sampling frame, randomly sampled 2,980 out of 10,535 health facilities in Kenya, but purposely included all secondary hospitals, public primary hospitals and maternity and nursing homes. Sixty one out of seventy four internship training centres were sampled in KHFA. The survey included over 3000 questions covering five main modules on resource availability, management and finance, readiness, quality and safety of health care, and data verification (information systems). Data were collected through facility audit, observation and validation, provider interview and record review. To supplement the KHFA data we extracted: the inpatient bed number in 2020 from the MFL (14); the total delivery count between 2018 to 2020 from the national Kenya Health Information System (previously District Health Information Software-2 [DHIS2]); and verified key human resources for health data for six hospitals with incomplete information through direct contact with hospitals.

Domains and indicators

We focus on the ‘structure’ component of the Donabedian quality of care framework to characterise hospitals. Specifically, we explore staffing, equipment and service availability and whether they fulfil the minimum requirement outlined in the Kenyan National Guidelines for Internship Training of Medical and Dental Officer Interns (2019) and the

For peer review only

For peer review only - http://bmjopen.bmj.com/site/about/guidelines.xhtml

National Guidelines and Log Book for Medical Officer Interns (2019) (2). For example, the guidelines require that each internship training centre: i) has a minimum of 5 medical specialists, covering paediatrics & child health, general surgery, internal medicine, obstetrics-gynaecology (OBGYN) and family medicine, ii) is fully operational on a 24-hour basis including accident & emergency, diagnostic and pharmacy services, iii) enables interns to observe and/or perform certain procedures to fulfill competency for each specialty. Examples of the latter in surgery include a total of 36 competency procedures such as appendicectomy or repair of inguinal hernias. This requires that these services be provided by internship training centres (2).

Two authors (ME, YZ) initially filtered out 277 indicators from the 3000 questions from KHFA to identify those most relevant to the minimum criteria defined by the national internship guidelines. Two authors (BO, FW) who are medical school faculty and who supervise medical officer interns subsequently reviewed these indicators identifying those felt to be most useful to characterizing hospitals as contexts for internship training by. A final list of 166 indicators was agreed by all four authors and selected for further analysis as "signal indicators" of the minimum requirements for internship training centres. All the indicators were converted into binary responses with 1 representing "met/available" and 0 representing "not met/unavailable". We grouped indicators into 12 logical domains described in brief in Table 1 with further detail of all indicators available in online supplement material 1.

For peer review only

173 ## Analysis

174 For each domain, we calculated a score as a percentage based on the number of indicators
175 with a score of 1 (met/available) divided by the total number of indicators (those
176 met/available + those not met/unavailable). We generated an overall readiness and
177 capacity index as the unweighted mean of all 12 domain scores for each facility. We
178 stratified the 61 included internship training centres into three categories based on hospital
179 administrative level and inpatient bed number. Kenya has a six service-level system based
180 on the essential package for health, whereas Level 1 refers to community-level, Level 2
181 dispensaires and clinics, Level 3 health centres, Level 4 primary facilities including district
182 hospitals, Level 5 secondary provicinal hospitals and Level 6 tertiary and national hospitals.
183 We first grouped all internship hospitals that are Level 5 & 6 together (bed number 174-
184 1455, n=15) as historically they have been better resourced; for the the internship hospitals
185 that are level 4, we divided them based on bed number into: Level 4 small hospitals (bed
186 number 82-176, n=23) and Level 4 large hospitals (bed number 176-320, n = 23). Data
187 analysis was conducted using Stata version 16 (Stata Corporation, Texas, USA).

188

189 ## Patient and public involvement

190 Patients and the public were not directly involved in the design, recruitment or conducting
191 of this study.

192

For peer review only - http://bmjopen.bmj.com/site/about/guidelines.xhtml

Results

Sixty one out of seventy four (61/74) internship training centres are sampled in KHFA and are mostly public hospitals owned by the Ministry of Health (n=54). The 13/74 hospitals not sampled are either private hospitals (n=4), faith-based or mission hospitals (n=8) or a military hospital (n=1). Comparing hospital characteristics retrieved from DHIS2 and MFL between the 61 KHFA-sampled hospitals with 12 of the 13 other hospitals (the military hospital did not report to DHIS2 or MFL), the 61 KHFA hospitals are slightly larger in terms of hospital beds (median and inter-quarter range [IQR] 200 [156, 276] vs. 180 [106, 221]), and report conducting three times as many deliveries (median annual deliveries 3981 vs. 1150) and twice as many Caesarean sections (median 987 vs. 537 per year). Detailed characteristics comparing the 61 KHFA internship training centres and these 12 others are presented in online supplement material 3.

Overall readiness and capacity

The distribution of capacity and readiness score by domain is presented in Figure 1 and Table 2. Ideally, an internship centre should score 100% against the (minimum) signal indicators but the overall capacity and readiness index is 69% (95%CI 66%-72%) for all internship training centres, and 63%, 67% and 81% for Level 4 small, Level 4 large and Level 5 & 6 hospitals respectively. Hospitals have moderate capacity and readiness for most of the general domains other than quality and safety. Surgery and OBGYN have higher scores regardless of hospital size, level and location, suggesting that the capacity and readiness for these two specialties is better across all facilities. Level 5 & 6 facilities have higher capacity scores in all domains when compared with Level 4 hospitals.

For peer review only

For peer review only - http://bmjopen.bmj.com/site/about/guidelines.xhtml

Human resources

The 61 internship training hospitals had a median of 14 post-licensure medical officers, 4 medical specialists, 25 clinical officers, 210 nurses and midwives, 14 medical laboratory technologists, 5 radiographers and 14 allied health professionals. Differences between small and large Level 4 hospitals were small for most cadres, whereas Level 5 & 6 hospitals have, on average, double the numbers of specialists, nurses and midwives (Table 3). The national guideline specifically requires at least 5 medical specialists to act as the main clinical teachers and supervisors for medical officer interns. However, only 29 out of 61 internship training centres had at least five medical specialists assigned, employed or seconded (including part-time staff). Most hospitals had at least one surgeon (n=59/61), paediatrician (n=52/61) and an obstetrician-gynaecologist (n=56/61), whereas only 35/61 hospitals have at least an internal medicine specialist. Although not a requirement from the regulator, only 30/61 hospitals have a physician anaesthesiologist (Figure 2) with anaesthesia mostly provided by clinical officers (51/61 hospitals have at least one anaesthetist clinical officer). The 4 well-established university teritary hospitals have better human resources and all but one fulfilled all the requirements.

Diagnostics and supportive care

As for laboratory tests, internship training centres overall have moderate availability (over 60%) for rapid tests and basic laboratory lab tests (Figure 3 and online supplement material 2), although ability to offer urine dipstick testing was present in fewer than 50% hospitals (n=26/61). 54/61 hospitals have rapid HIV tests, 16 hospitals provided onsite polymerase chain reaction (PCR) testing for HIV but only 5 had onsite HIV viral load testing. An additional

For peer review only - http://bmjopen.bmj.com/site/about/guidelines.xhtml
For peer review only

19 hospitals relied on off-site PCR testing. Availability for advanced lab tests is poor across all hospitals, only 12/61 hospitals can read papanicolaou test (PAP smear) results and prepare and examine any tissues or samples for cancer diagnosis onsite. Level 5 and 6 hospitals on average have better availability of diagnostics than Level 4 hospitals, especially for infectious disease and advanced lab tests.

Internship training hospitals vary when assessed for 10 oxygen and respiratory support indicators. While all hospitals were reported to have functional paediatric and neonatal oxygen, only 32 hospitals have ventilators available for adults and 13 hospitals had continuous positive airway pressure (CPAP) available for neonates (at the time of survey in 2018 pre-Covid-19). Lastly for other general equipment, around 55 hospitals reported functioning ultrasound and X-ray services. In comparison, functional electrocardiogram (ECG) (27/61) and computerised tomography (CT) scan (21/61) availability was poor in most facilities.

Service continuity and safety

The national guideline requires that internship hospitals need to be fully operational on a 24-hour basis including accident & emergency, laboratory diagnostic services, and pharmacy which was investigated under “24/7 availability”. 48/61 hospitals reported having general medical officers or emergency medicine specialists onsite or on-call within facility. While emergency radiology, laboratory diagnostics (other than rapid test) and pharmacy services are available 24/7 in most facilities, only 13/61 hospitals reported availability of major emergency surgery and anesthesia 24/7, and only 16 hospitals provide 24-hour emergency services using a structured triage tool such as the WHO integrated triage tool.

For peer review only

For peer review only - http://bmjopen.bmj.com/site/about/guidelines.xhtml

The domain average score for infection prevention and control is 66%. Most internship training centres have infection prevention and control (IPC) guidelines and technical IPC committees, however, the actual procedures of cleaning beds, counters/tables, toilets were only done in around half of the hospitals on the day of KHFA survey observation. Twenty one input/structure indicators on quality and safety are also included in the current analysis. All but one indicator (death review results recorded) performed poorly across all facilities. Most facilities do not have observed guidelines for adverse event reporting, nosocomial infection reporting, surgical adverse event reporting or postoperative infection definitions. Level 5 & 6 hospitals do not necessarily perform better than Level 4 hospitals in this domain.

Major specialties

For the four major specialties we used data on both specialty-specific equipment & medicines and specialty service areas (online supplement material 2). For surgical and OBGYN scores, internship training centres in general have both functional equipment & available medicine (median 100% for surgery, 100% for OBGYN) and also good service availability for procedures relevant to the national guideline and log book (median 100% for surgery, 90% for OBGYN). The only indicator that had a lower score is cricothyroidotomy, where only 43/61 hospitals provide such service; and misoprostol tablet availability, where 44/61 hospitals have this medication available.

For internal medicine, availability of equipment and medicine is low (median 67%) with huge variation across facilities. 33/61 hospitals have a defibrillator, 39/61 have a renal dialysis/hemodialysis machine and 23/61 hospitals have lumbar puncture kits – for the rest

For peer review only - http://bmjopen.bmj.com/site/about/guidelines.xhtml

of the hospitals they will need to improvise to perform lumbar punctures or cannot provide such a service. Service availability for internal medicine is good on average (median 90%), however availability for more advanced conditions e.g. colorectal cancer screening, diagnosis and/or treatment, and palliative care is limited especially for Level 4 small hospitals. Similar with internal medicine, the availability of equipment for paediatrics is lower as compared with other specialties (median 63%). Only 19/61 hospitals had exchange blood transfusion capacity and similarly only 19/61 hospitals had devices for intraosseous (IO) access. The three indicators of paediatric service availability (kangaroo mother care, sepsis, antibiotics regimen and follow-up) had good availability.

Medical officer interns are also required to rotate in mental health for eight weeks during their one-year internship and are required to manage acute mental health emergencies and common mental health conditions. While 52/61 hospitals offer mental health and/or neurological services, only 17 hospitals have a physician psychiatrist. Sixteen hospitals have mental health inpatient wards, other hospitals either only provide outpatient services or lack these services. Only half of Level 4 small hospitals offer depression, psychosis and epilepsy diagnosis and follow-up services.

Discussion

Our analysis on the readiness and capacity of Kenya internship training hospitals suggested that in 2018 the overall staffing, equipment and service availability was inadequate when compared with the regulator's requirements. Level 4/district, smaller hospitals (23 of the 61 assessed) were more likely to have fewer human resources, equipment and specialty

For peer review only

services, and less likely to meet quality and safety indicators. The implication is that hospitals with lower capacity may either have to improvise to offer core services or refer patients to other facilities. This could significantly influence the quality of training as interns might not be able to fully consolidate their knowledge and skills.

Over half of the internship training hospitals did not have five or more specialists as required by the regulator, the medical council, which suggests inadequate training and supervision of medical officer interns. Internal medicine specialists and anaesthesiologists were most often lacking suggesting that in many centres non-specialist but licensed general medical or specialist clinical officers fill these roles. Even in these larger Kenyan hospitals such personnel will also likely therefore be leading provision of care for severe COVID-19. Where medical officers are themselves also scarce, medical officer interns may become the only “in-house” doctor on call and be forced to take on full responsibility for patient care potentially threatening patient safety if interns are forced to care for serious, acute medical and surgical conditions without appropriate supervision (9,15,16). The likely challenges to provision of high quality safe care are further suggested by poor scores of all but tertiary hospitals against organisational indicators of quality and safety practice. In fact guidelines or instruction for quality and safety were rarely present and there seemed very limited evidence that quality and safety indicators are monitored, reviewed, reported and acted upon. These findings are consistent with reports highlighting challenges with patient safety in low- and middle-income country (LMIC) hospitals (17).

Internship hospitals appear most able to provide appropriate essential resources and capacities in the major disciplines of surgery and OBGYN. Challenges were especially noted

For peer review only

in the disciplines of internal medicine and paediatrics and neonatology. Many facilities lack ECG machines, CT scans, defibrillators, equipment used for peritoneal or hemo-dialysis, and lumbar puncture kits. More specific to neonatal and child health were lack of capacity in ability to provide exchange transfusion, neonatal CPAP and intraosseous access. Capacity to offer mental health and neurological care, only listed as internship requirements in 2020, was especially low most particularly in Level 4 small hospitals. Kenya has a severe shortage of psychiatrists and neurologists as well as psychiatric nurses (18). For facilities without capacity in the major disciplines now including mental health and neurology services it is likely that hospitals are forced to refer patients elsewhere or improvise approaches to care in order to serve patients. Interns' training experience is therefore likely to be very varied and more attention may therefore need to be paid to planning how interns can be provided with adequate training in all specialties if this is a key long-term aim.

Kenya has dramatically increased its medical training volume hoping to produce 9,000 new graduate medical physicians by 2030 to narrow its staffing gap (6). More hospitals especially at county-level (district hospitals) are accredited as internship training centres. These hospitals are usually smaller in size and more likely to be distant from the well-established university tertiary hospitals in major cities that scored much higher across domians in our analysis. Rural rotations and residencies have been recommended by the WHO to increase health worker retention (19). Students or interns in these settings may also be more likely to learn hands-on clinical procedures and be actively engaged in patient care (20) as opposed to tertiary hospitals where the presence of more specialists and general medical officers may result in interns undertaking more administrative work. However, the prerequisite of good internship training is that the hospitals are adequately staffed and equipped and ready

For peer review only

to deliver teaching and training. Our data suggested that this is not always the case and are consistent with previous qualitative research suggesting that district hospitals provided limited learning with limited suitability as internship sites (9).

The potential consequences of poor training and supervision during internship are therefore worth consideration. As well as failing to consolidate knowledge and skills in major specialties interns may also develop burnout and stress-related psychological problems more rapidly (9). These stresses and being forced to take significant clinical responsibility in poorly-resourced hospitals may push medical officer interns to leave the profession or the public sector which is often most resource constrained as soon as they are licensed/registered (21). This will likely worsen the internal and external brain-drain undermining universal health coverage and equity and limiting any benefits from public investment in medical education (21). Inadequate support and supervision also threatens patient safety by creating the conditions for significant medical errors to occur. Even in settings without a 'blame culture' such errors can have profound effects on the health workers as well as the patients further exacerbating workers' psychological distress (22,23). We must also remember that todays' medical officer interns will become tomorrows general medical officers who are often then responsible for the training of the next cohorts of interns and other health worker cadres (24). Therefore, poor internship training for medical officers continuously compromises the quality of training and skills that are passed on and this may be a particular challenge to patient safety and healthcare quality that rely so heavily on effectively functioning teams (25).

For peer review only

For peer review only - http://bmjopen.bmj.com/site/about/guidelines.xhtml

Our analyses have clear implications for Kenya policy-makers. The regulator requires internship hospitals to maintain the minimum requirement of staff and be able to offer a core set of quality services with self-report against such requirements at least once a quarter. Our data would suggest many internship hospitals in Kenya might not continuously achieve these minimum requirements. Should this prevent interns from being licensed or should interns be reallocated to centres meeting regulatory requirements? While more stringent and regular audit and re-accreditation of internship training centres might be conducted by the regulatory council to ensure that only the hospitals that meet the minimum requirements are allowed to receive and train interns, adequate mitigation measures need to be in place so that interns themselves are not disadvantaged further. More widely, our findings point to the need to carefully consider the potential consequences of rapidly expanding medical training and of appropriate planning and financing for new internship centres, especially in rural areas.

Several limitations should be noted for the current analysis. To start with, our analysis was only limited to the 61 internship training hospitals sampled by KHFA, mostly public hospitals. A total of 13 hospitals were not sampled in KHFA and are either private hospitals or mission hospitals, therefore comparison by hospital ownership was not feasible. Second, aside from the KHFA's own limitation on data missing, we noted data inaccuracy and inconsistency in KHFA data we retrieved. We made efforts to clean these data especially on human resources through correspondence input though we were unable to validate all the number from the KHFA survey. Third, to assess the readiness and capacity of internship training hospitals we selected 166 signal indicators from the 3000 questions from KHFA survey. Our criteria were to ensure that the indicators selected are the minimum requirement and

For peer review only

should be achievable (i.e. should be “1” for all indicators). Our selection was guided by the national guidelines and agreed by four authors, three of whom have experience supervising interns. However, we acknowledge that this process is somewhat subjective. Fourth, we only focused on the structural and organizational features of internship training hospitals. We did not include indicators on process or outcome indicators e.g. training or patient outcomes due to limited data availability. Ideally these should be considered while evaluating internship training hospitals. Last but not least, the KHFA data was collected in a snapshot in 2018 prior to Covid-19. The appointment and posting of specialists may fluctuate from time to time but Covid-19 specifically could have led to the government investing in hospital infrastructure, equipment and emergency hiring to improve their response capacity to Covid. Therefore it is possible that the readiness and capacity of Kenyan internship training centres has improved since 2018 (26). Despite these limitations our data do suggest important shortcomings in internship training centres in Kenya. We suggest other LMICs that are rapidly expanding their medical training should also evaluate their internship training sites to explore the generalisability of our findings.

For peer review only

Conclusion

We assessed the readiness and capacity of 61 Kenyan internship training hospitals using data from KHFA 2018. Our results highlighted major gaps in staffing, equipment and service availability with particular weaknesses in areas exploring organisational arrangements to support quality and safety. Smaller hospitals are more likely to have a lower readiness and capacity index, and should be given much greatrer support so they can provide appropriate

and well-resourced training for medical interns allowing them to become fully competent medical doctors.

List of abbreviations

CPAP, continuous positive airway pressure

CT, computerised tomography

DHIS2, District Health Information Software-2

IPC, infection prevention and control

IQR, inter-quarter range

KHFA, Kenya Harmonized Health Facility Assessment

LMIC, low- and middle-income country

MFL, Kenya Health Master Facility List

OBGYN, obstetrics and gynaecology

PCR, polymerase chain reaction

Footnote

Online supplemental material 1. 166 Indicators included for each general domain and speciality domain

Online supplemental material 2. All indicator heatmap by hospital bed number

Online supplemental material 3. Comparing internship hospitals sampled in KHHFA and others

Online supplemental material 4. STROBE statement and checklist

Acknowledgements: Not applicable.

For peer review only

Funding: YZ is supported by the University of Oxford Clarendon Fund Scholarship. ME is supported by a Wellcome Trust Senior Research Fellowship (#207522). HK is employed by the Kenya Ministry of Health.

Competing interests: The authors declare that they have no competing interests.

Patient consent for publication: Not applicable.

Authors' contributions: YZ and ME conceived of the analysis. YZ, BO, FW and ME contributed to data analysis. YZ wrote the first draft of the manuscript. BO, FW, HK, DG, CN and ME provided critical feedback on the first draft of the manuscript. All authors read and approved the final manuscript.

Data sharing statement: The data that support the findings of this study are available from Kenya Ministry of Health but restrictions apply to the availability of these data, which were used under license for the current study, and so are not publicly available. All requests for further use of these data can be made through and with the permission of the Kenya Ministry of Health.

Ethics approval and consent to participate: The Kenya Ministry of Health, Division of Monitoring and Evaluation, sought approval for, planned and execute the 2018 Kenya Harmonized Health Facility Assessment. Written informed consent was sought by in-charge or acting in-charge of all the facilities surveyed. Permission was then granted to use these data for the secondary analysis reported here with the purpose of improving quality of health service provision in Kenya.

For peer review only

For peer review only - http://bmjopen.bmj.com/site/about/guidelines.xhtml

Reference

1. Ndetei DM, Mathai M, Khasakhala LI, Mutiso V, Mbwayo AW. University medical education in Kenya: The challenges. Medical Teacher. 2010 Oct 1;32(10):812–5.

2. MP&DC. National guidelines and log book for medical officer interns [Internet]. Available from: http://medicalboard.co.ke/resources/NATIONAL%20GUIDELINES%20AND%20LOG%20BOOK%20FOR%20MEDICAL%20OFFICER%20INTERNS.pdf

3. Kupersmith J. Quality of care in teaching hospitals: a literature review. Academic Medicine. 2005;80(5):458–66.

4. COLWELL NP. THE REQUIREMENT OF AN INTERN HOSPITAL. Journal of the American Medical Association. 1929 Mar 30;92(13):1031–3.

5. Ogero M, Akech S, Malla L, Agweyu A, Irimu G, English M, et al. Examining which clinicians provide admission hospital care in a high mortality setting and their adherence to guidelines: an observational study in 13 hospitals. Arch Dis Child. 2020;105(7):648–54.

6. English M, Strachan B, Esamai F, Ngwiri T, Warfa O, Mburugu P, et al. The paediatrician workforce and its role in addressing neonatal, child and adolescent healthcare in Kenya. Archives of Disease in Childhood. 2020 Oct 1;105(10):927–31.

7. Kenya Ministry of Health. Kenya Health Workforce Report: The Status of Healthcare Professionals in Kenya, 2015. [Internet]. 2017. Available from: https://taskforce.org/wp-content/uploads/2019/09/KHWF_2017Report_Fullreport_042317-MR-comments.pdf

8. Kenya Ministry of Health. Kenya Human Resource for Health Strategic Plan 2019-2023. 2019.

9. Muthaura PN, Khamis T, Ahmed M, Hussain SR. Perceptions of the preparedness of medical graduates for internship responsibilities in district hospitals in Kenya: a qualitative study. BMC Med Educ. 2015 Oct 21;15:178.

10. Approved Internship Centres- Kenya Medical Practitioners and Dentists Council [Internet]. [cited 2020 Feb 12]. Available from: https://medicalboard.co.ke/functions/approved-internship-centres/

11. Njiri F. Strengths And Challenges Of Decentralized Health Facilities As Medical Training And Research Sites In Kenya. 2013 Jun [cited 2021 Mar 11]; Available from: http://erepository.uonbi.ac.ke/handle/11295/61812

12. Muasya MK, Dienya TM, Wagaiyu EG, Nduati R, Kiarie JN. Capacity of non-tertiary Kenyan health facilities selected for decentralised dental training. East African Medical Journal. 2016;93(2):55–9.

For peer review only

13. Kenya Ministry of Health. Kenya Health Facility Assessment (KHFA) 2018/19 Reports [Internet]. [cited 2021 Mar 9]. Available from: https://www.health.go.ke/resources/downloads/

14. Kenya Master Health Facility List: Find all the health facilities in Kenya [Internet]. [cited 2021 Mar 9]. Available from: http://kmhfl.health.go.ke/#/home

15. Capinpuyan ALC, Miguel RTD. Ethical Challenges in Medical Community Internships. Health Hum Rights. 2019 Jun;21(1):141–7.

16. Bola S, Trollip E, Parkinson F. The state of South African internships: A national survey against HPCSA guidelines. S Afr Med J. 2015 Sep 21;105(7):535.

17. Wilson RM, Michel P, Olsen S, Gibberd RW, Vincent C, El-Assady R, et al. Patient safety in developing countries: retrospective estimation of scale and nature of harm to patients in hospital. BMJ. 2012 Mar 13;344:e832.

18. Meyer A-C, Ndetei D. Providing Sustainable Mental Health Care in Kenya: A Demonstration Project [Internet]. Providing Sustainable Mental and Neurological Health Care in Ghana and Kenya: Workshop Summary. National Academies Press (US); 2016 [cited 2021 Mar 12]. Available from: https://www.ncbi.nlm.nih.gov/books/NBK350312/

19. Increasing Access to Health Workers in Remote and Rural Areas Through Improved Retention: Global Policy Recommendations [Internet]. Geneva: World Health Organization; 2010 [cited 2020 Feb 25]. (WHO Guidelines Approved by the Guidelines Review Committee). Available from: http://www.ncbi.nlm.nih.gov/books/NBK138618/

20. Kibore MW, Daniels JA, Child MJ, Nduati R, Njiri FJ, Kinuthia RM, et al. Kenyan medical student and consultant experiences in a pilot decentralized training program at the University of Nairobi. Educ Health (Abingdon). 2014 Aug;27(2):170–6.

21. Essa ZI. Post-medical interns' reflections on medical internships in South African state training hospitals. [Internet] [Thesis]. 2011 [cited 2021 May 11]. Available from: http://wiredspace.wits.ac.za/handle/10539/9209

22. Goddard AF, Patel M. The changing face of medical professionalism and the impact of COVID-19. The Lancet. 2021 Mar 13;397(10278):950–2.

23. Wu AW, Steckelberg RC. Medical error, incident investigation and the second victim: doing better but feeling worse? BMJ Qual Saf. 2012 Apr 1;21(4):267–70.

24. Kenya Scheme of service for medical officers' dental officers and pharmacists [Internet]. 2017. Available from: https://www.cog.go.ke/media-multimedia/reportss/category/101-schemes-of-service?download=260:scheme-of-service-for-medical-officers-dental-officers-and-pharmacists

For peer review only

25. Doctors may learn bad habits at teaching hospitals with safety violations [Internet]. STAT. 2018 [cited 2021 Apr 3]. Available from: https://www.statnews.com/2018/04/20/doctors-learn-bad-habits-teaching-hospitals/

26. Barasa EW, Ouma PO, Okiro EA. Assessing the hospital surge capacity of the Kenyan health system in the face of the COVID-19 pandemic. PLoS One [Internet]. 2020 Jul 20 [cited 2021 May 11];15(7). Available from: https://www.ncbi.nlm.nih.gov/pmc/articles/PMC7371160/

For peer review only

Table 1. Domain and indicator summary

Category	Domain	Number of indicators	Example indicators	Indicator and domain conversion
Human resources	(a) Human resource for health	6	q251c_04 & q251c_23: Total staff assigned, employed, seconded (including part time) – Paediatricians, Neonataologists	All the indicators were converted into binary responses with 1 representing "available" and 0 representing "unavailable". For most indicators on availability, we defined available as "onsite, observed, non-expired and functional". For tests that are reported to be available offsite, or tests that are observed but expired, and for equipment reported available but not observed, or not available now, we labeled them as "unavailable". We also considered an indicator to be unavailable if the health facility did not answer this question, usually because its filter questions were answered no.
Diagnostics and supportive care	(b) Laboratory test (including 4 sub-domains, rapid test, basic lab test, infectious diseases test, advanced lab test)	31	q6307a_6: TB test availability - Xpert MTB/RIF rapid diagnostic testing for tuberculosis	
	(c) Oxygen and respiratory support	10	q2250b_10: Please tell me if the pulse oximeter are available anywhere in the outpatient service area and are functional.	
	(d) General equipment	8	q4981b_1: Electrocardiogram (ECG) Equipment available and functioning today	
Service continuity and safety	(e) 24/7 availability	8	Q4885a: Is emergency medicine specialist or general medical practitioners always available 24-hours for emergency service, either onsite in emergency unit or not onsite in emergency unit but on-call inside facility?	
	(f) Infectious prevention and control	9	q7220: Does this facility have guidelines or protocols for cleaning the facility such as the floors, counters, and beds?	
	(g) Quality and safety	24	K7172: How frequently does the quality assurance committee meet? - Monthly	
Major specialties	(h) Surgery (including 2 sub-domains, equipment/medicine and service)	20	q5113b_07: Functioning of basic surgical equipment - EKG electrodes	
	(i) Internal medicine (including 2 sub-domains, equipment/medicine and service)	12	q2615: Do providers in this facility diagnose and/or manage diabetic patients?	
	(j) Obstetrics-gynaecology (including 2 sub-domains, equipment/medicine and service)	11	q4515a_1: Parenteral administration of antibiotics (IV or IM) for mothers carried out	
	(k) Paediatrics (including 2 sub-domains, equipment/medicine and service)	20	q4660: Is KMC (Kangaroo mother care) for premature/very low birth-weight babies) used in this facility?	
	(l) Mental health	6	q2700: Does this facility offer any services for mental and/or neurological conditions?	

For peer review only

For peer review only - http://bmjopen.bmj.com/site/about/guidelines.xhtml

Table 2. Capacity and readiness scores by hospital characteristics

	Level 4 small hospitals (n=23)	Level 4 large hospitals (n=23)	Level 5 & 6 hospitals (n=15)	Total (n=61)
Total capacity & readiness index	63%	67%	81%	69%
Human resources for health	64%	67%	89%	71%
Lab test	60%	63%	77%	65%
Oxygen & respiratory support	64%	57%	77%	64%
General equipment	53%	59%	72%	60%
24/7	58%	65%	68%	63%
Quality and safety	35%	38%	50%	40%
Infection prevention & control	57%	58%	92%	66%
Surgery	94%	89%	97%	93%
Internal medicine	70%	73%	90%	76%
Paediatrics	67%	70%	81%	72%
OBGYN	89%	91%	91%	90%
Mental health/Neurology	44%	73%	83%	64%

Table 3. Human resources for health cadres by hospital characteristics

	Level 4 small hospitals (n=23)	Level 4 large hospitals (n=23)	Level 5 & 6 hospitals (n=15)	Total (n=61)
Medical officer	12 (6, 18)	14 (10, 20)	29 (21, 58)	14 (10, 26)
Medical specialist	2 (1, 7)	4 (1, 11)	16 (2, 26)	4 (1, 11)
Clinical officer	20 (13, 26)	25 (15, 33)	35 (21, 44)	25 (15, 33)
Nurse and midwife	206 (95, 248)	150 (113, 210)	336 (267, 534)	210 (114, 323)
Medical laboratory technologist	13 (11, 14)	14 (11, 18)	23 (19, 46)	14 (12, 20)
Radiographer	4 (3, 5)	4 (2, 6)	9 (6, 22)	5 (3, 7)
Allied health professional	11 (3, 14)	14 (10, 17)	29 (24, 44)	14 (6, 24)

Note: Data are presented as median (interquartile range), allied health professional refers to physical therapist, occupational therapist, orthopedic technician, paster technician, nutritionists and dieticians

For peer review only

Figure legends

Figure 1. Distribution of capacity and readiness score by domain

Figure 2. Number of internship training centres with different numbers of specialists (a) and with no surgeon, internists, obstetrician-gynaecologist, paediatrician and anaesthesiologist (b)

Note: We have combined different types of surgeons (general surgeons, paediatric surgeons, orthopaedic surgeons, cardiothoracic surgeon, ENT surgeons, plastic surgeons, neuro-surgeons, urological surgeons) into "surgeon" category. Similarly, specialist physicians (internists), oncologist, neurologist, cardiologist, critical care, gastroentologist, palliative care specialist, nephrologists, rheumatologist, medical endocrinologist into "internist" category, and paediatricians and neonatologists into "paediatrician" category.

Figure 3. Average lab test score by sub-domain and hospital characteristics

For peer review only

For peer review only - http://bmjopen.bmj.com/site/about/guidelines.xhtml

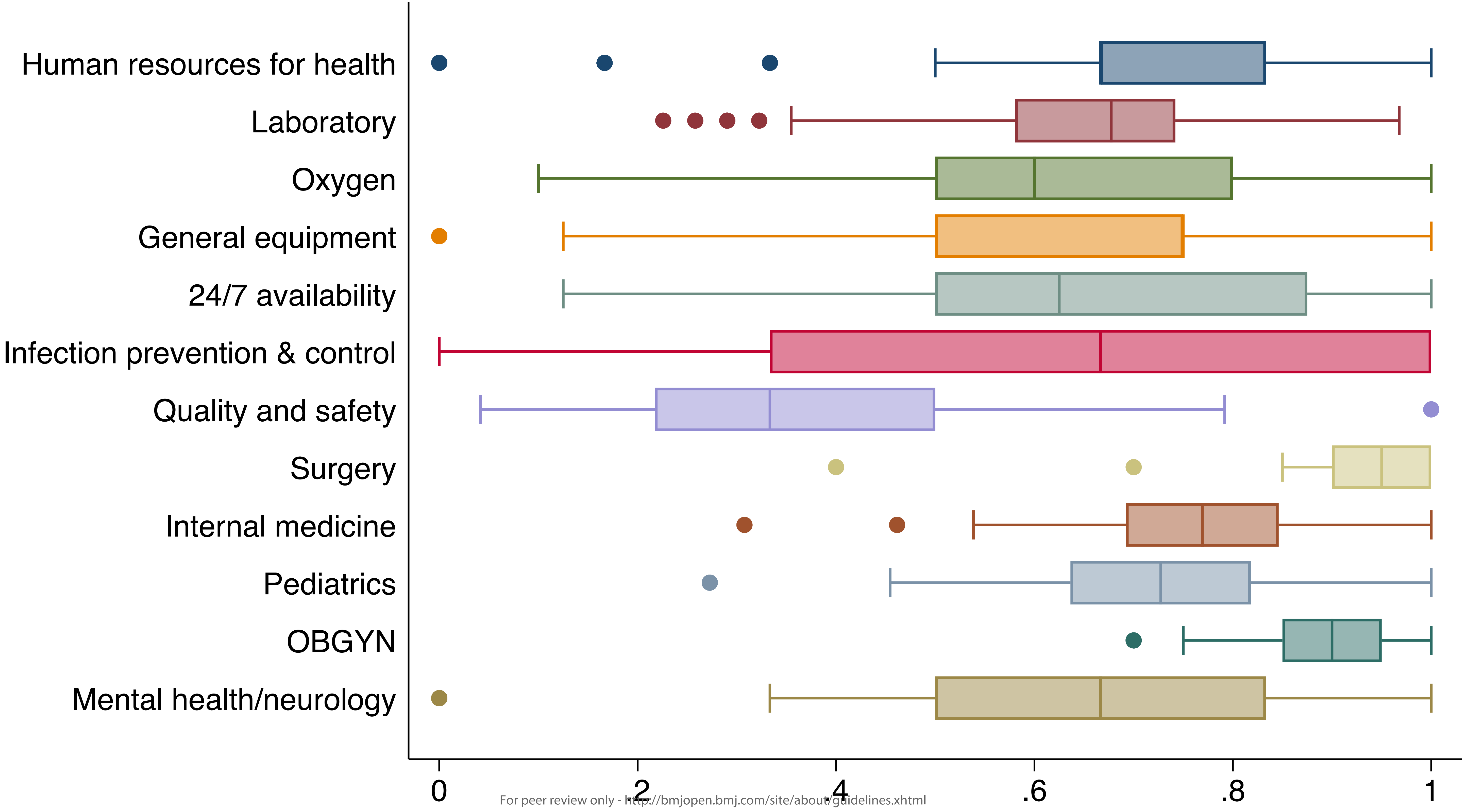
Human resources for health
Laboratory
Oxygen
General equipment
24/7 availability
Infection prevention & control
Quality and safety
Surgery
Internal medicine
Pediatrics
OBGYN
Mental health/neurology
0
.2
.4
.6
.8
1

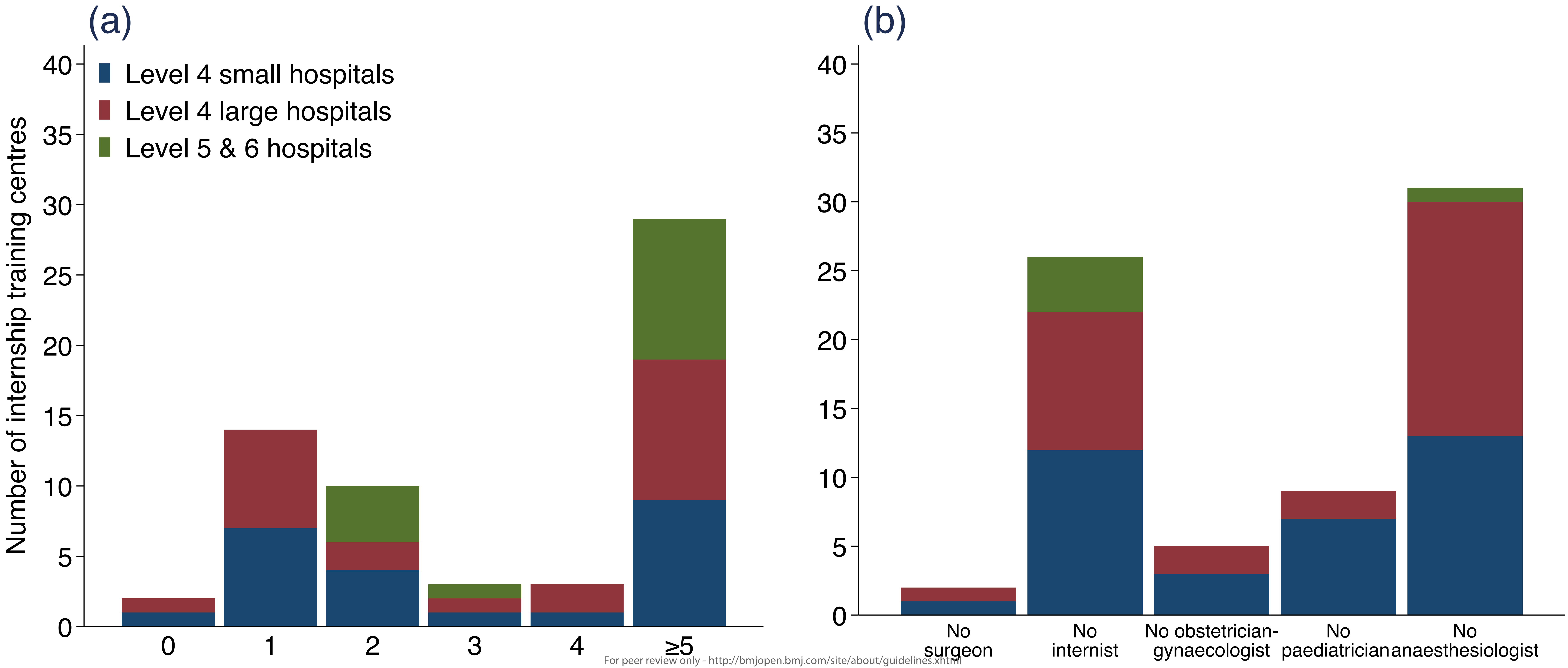
(a)
Level 4 small hospitals
Level 4 large hospitals
Level 5 & 6 hospitals
Number of internship training centres
0
5
10
15
20
25
30
35
40
0
1
2
3
4
≥5
(b)
0
5
10
15
20
25
30
35
40
No surgeon
No internist
No obstetrician-gynaecologist
No paediatrician
No anaesthesiologist

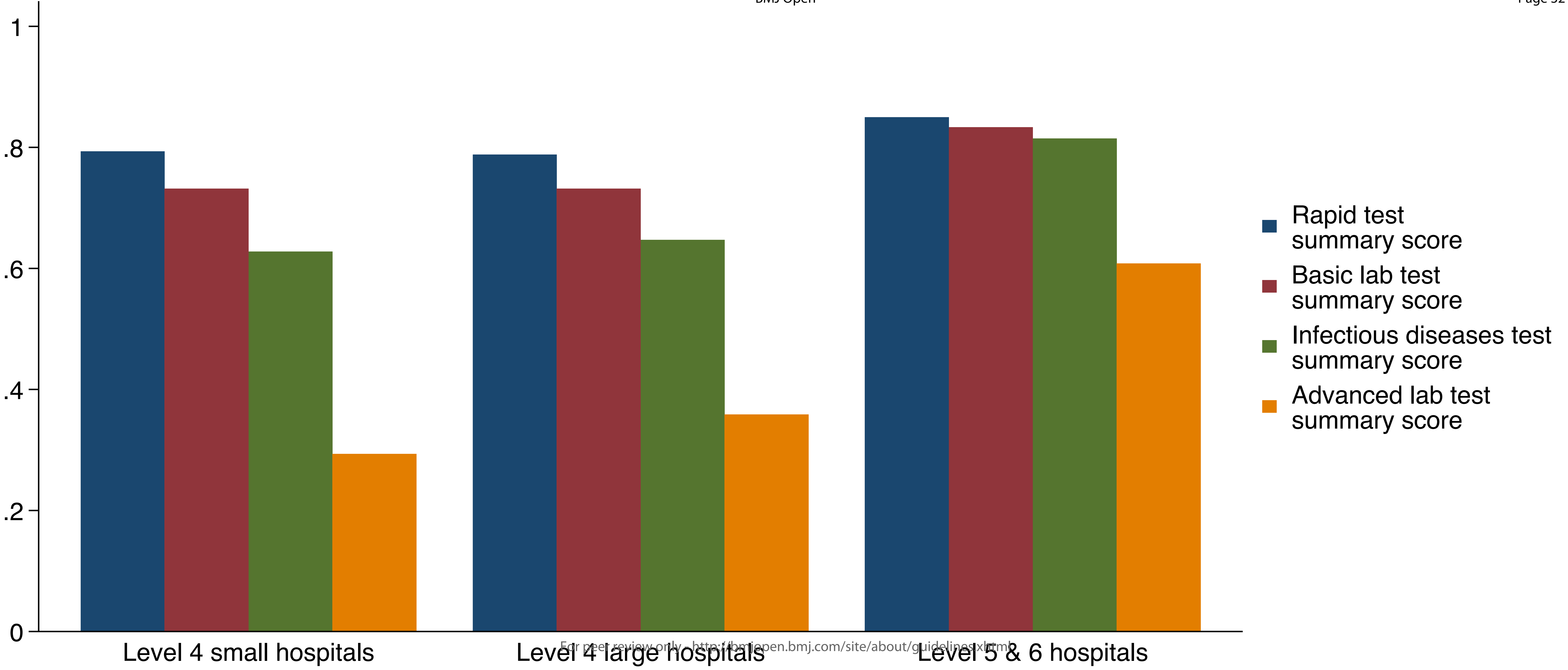

For peer review only - http://bmjopen.bmj.com/site/about/guidelines.xhtml

Additional file 1. 166 Indicators included for each general domain and speciality domain

Domain	Indicator	1 – considered available	0 – considered unavailable
**Human resources for health (6)**	At least five medical specialists	- 5 or more specialists	- Less than 5 specialists
	At least one surgeon	- 1 or more	- Less than 1
	At least one internist	- 1 or more	- Less than 1
	At least one paediatrician	- 1 or more	- Less than 1
	At least one obstetrician-gynaecologist	- 1 or more	- Less than 1
	At least one anaesthesiologist	- 1 or more	- Less than 1
**Lab test (31)**	HIV rapid test	- Observed, at least one not expired	- Not observed, reported available but not seen<br>- Not observed, never available
	Syphilis rapid test	- Observed, at least one not expired	- Not observed, reported available but not seen<br>- Not observed, never available
	Urine rapid test for pregnancy	- Observed, at least one not expired	- Not observed, reported available but not seen<br>- Not observed, never available
	Urine dipstick 3	- Observed, at least one not expired	- Not observed, reported available but not seen<br>- Not observed, never available
	Haemoglobin A1c rapid test	- Observed, at least one not expired	- Not observed, reported available but not seen<br>- Not observed, never available
	Colorimeter or haemoglobinometer	- Observed, functional/valid	- Reported (not seen), functional/valid<br>- Not available today
	Glucometer	- Observed, functional/valid	- Reported (not seen), functional/valid<br>- Not available today
	Glucometer test strips/discs	- Observed, functional/valid	- Reported (not seen), functional/valid<br>- Not available today
	Any blood chemistry test	- Yes, onsite	- No, never available
	Renal function test	- Yes, observed, functional/valid	- No, not available today
	Other tests for bilirubin	- Yes, onsite	- Yes, offsite (specimen sent)<br>- No, never available
	ABO blood grouping testing	- Yes, onsite	- Yes, offsite (specimen sent)<br>- No, never available
	Rhesus blood grouping testing	- Yes, onsite	- Yes, offsite (specimen sent)<br>- No, never available
	Cross-match testing	- Yes, onsite	- Yes, offsite (specimen sent)<br>- No, not available today
	Onsite PCR for HIV viral load or HIV early-infant diagnosis	- Yes, onsite	- Yes, offsite (specimen sent)<br>- Yes, not fucntional/not valid<br>- No, not available today
	Xpert MTB/RIF rapid diagnostic testing for TB	- Yes, onsite	- Yes, offsite (specimen sent)<br>- No, not available today
	Urine microscopy	- Yes, observed, functional/valid	- No, not available today
	Malaria smears	- Yes, observed, functional/valid	- No, not available today
	Microscopy test CSF body fluid counts	- Yes, onsite	- Yes, offsite (specimen sent)<br>- No, never available
	Cryptococcal antigen testing	- Yes, onsite	- Yes, offsite (specimen sent)<br>- No, never available
	Gram stain testing	- Yes, onsite	- Yes, offsite (specimen sent)<br>- No, never available
	Culture and sensitivity test	- Yes, onsite	- Yes, offsite (specimen sent)<br>- No, never available
	Blood cultures test	- Yes, onsite	- Yes, offsite (specimen sent)<br>- No, never available
	Fine needle aspiration cytology	- Perform in facility	- Refer for test<br>- Not used
	Core needle biopsy of lump specimen	- Perform in facility	- Refer for test<br>- Not used
	Prostate Specific Antigen (PSA) Testing	- Perform in facility	- Refer for test<br>- Not used
	Blood gas measurement	- Yes, onsite	- Yes, offsite (specimen sent)

For peer review only

			- No, never available
	Cardiac marker (CK, Troponin) test	- Yes, onsite	- Yes, offsite (specimen sent)<br>- No, never available
	PAP smear read in facility	- Yes, read in facility	- Yes, read outside facility<br>- No<br>- 0 PAP smears were conducted with results recorded during the past 12 completed months
	VIA/VILLI read in facility	- Yes, read in facility	- Yes, read outside facility<br>- No<br>- 0 VIA/VILLI were conducted with results recorded during the past 12 completed months
	Prepare and examine tissues or samples for cancer	- Yes	- No
**Oxygen and respiratory support (10)**	Surgery oxygen equipment functioning - Oxygen tank/cylinder with attached pressure gauge, pressure regulator	- Yes	- No<br>- Don't know
	Maternity paediatric-sized oxygen delivery apparatus functioning	- Yes	- No
	Outpatient pulse oximeter functioning	- Yes	- No
	Maternity pulse oximeter functioning	- Yes	- No
	Paediatrics/neonatal oxygen functioning	- Yes	- No
	Emergency pulse oximeter functioning	- Yes	- No
	Ventilator available	- Yes, available now	- No
	Chest tubes available	- Observed, at least one not expired	- Not observed, not available today<br>- Not observed, never available
	Adult CPAP equipment available	- Observed, at least one not expired	- Observed, available but expired<br>- Not observed, reported available but not seen<br>- Not observed, not available today<br>- Not observed, never available
	Emergency paediatrics and neonatal CPAP available	- Observed	- Reported, not seen<br>- Not available
**General equipment (8)**	ECG offered	- Yes	- No
	ECG equipment functioning	- Yes equipment available and functioning	- No equipment not available or not functioning
	Ultrasound offered	- Yes	- No
	Ultrasound equipment functioning	- Yes equipment available and functioning	- No equipment not available or not functioning
	X-ray offered	- Yes	- No
	X-ray equipment functioning	- Yes equipment available and functioning	- No equipment not available or not functioning
	CT scan offered	- Yes	- No
	CT scan equipment functioning	- Yes equipment available and functioning	- No equipment not available or not functioning
**24 7 (8)**	Emergency service with structured triage tool 24/7	- 24-hour emergency service	- 10 hours<br>- No structured triage tool
	Medical officer onsite or oncall in facility 24/7	- MO or Emergency practitioner always available, onsite<br>- MO or Emergency practitioner always available, not onsite but oncall in facility	- MO or Emergency practitioner always available, not in facility<br>- Staff not available 24 hours
	Formal triage tool used 24/7	- Yes	- No
	Emergency surgery and anaesthesia 24/7	- 24-hour emergency service	- 0, 2 or 8 hours
	Emergency radiology 24/7	- 24-hour emergency service	- 0, 5, 9, 10, 12 hours
	Emergency diagnostics 24/7	- 24-hour emergency service	- 0, 7, 10, 12 hours
	Emergency pharmacy 24/7	- 24-hour emergency service	- 8, 9, 10, 12, 15 hours<br>- No dedicated pharmacy in facility
	Blood transfusion	- Yes available now	- No
**IPC (9)**	Facility has IPC guideline	- Yes, observed	- Reported not seen<br>- No
	Technical IPC committee	- Yes	- No
	Dedicated (full-time) IPC staff	- Yes	- No

For peer review only

	Guidelines for cleaning	- Yes, observed	- Reported not seen<br>- No
	Step-by-step techniques for specific tasks	- Yes	- No
	Cleaning roster or schedule specifying responsibility	- Yes	- No
	Cleaning beds observed	- Yes	- No
	Cleaning counters/tables observed	- Yes	- No
	Cleaning toilets observed	- Yes	- No
**Quality and safety (24)**	Monthly quality assurance committee meeting	- Monthly	- Quarterly<br>- Biannually
	Documentation of quality assurance information reviewed	- Yes, documentation observed	- Yes, documentation reported not seen
	Ongoing quality improvement using Plan-Do-Check-Study-Act approach	- Yes, service-specific<br>- Yes, across facility	- No
	Multi-disciplinary quality improvement team	- Yes	- No
	Departmental specific work improvement teams	- Yes	- No
	Documentation of improvement achieved as a result of quality improvement process	- Yes, documentation observed	- Yes, documentation reported not seen<br>- No
	Kenya Quality Model for Health completed in 2017 or 2018	- 2017<br>- 2018	- 2014<br>- 2016<br>- Don't know
	Formal case reviews carried out at least monthly	- At least weekly<br>- At least monthly	- At least quarterly<br>- No specific timing
	Death reviews results recorded	- Yes	- No
	Guidelines of events that are considered adverse and required to be reported	- Yes, documentation observed	- Yes, documentation reported not seen<br>- No
	Notes or reports that show evidence of review and plan of action for the reports about adverse events	- Yes, documentation observed	- Yes, documentation reported not seen<br>- No
	Written guidelines that define nosocomial infections and the process for reporting	- Yes, observed	- Reported not seen<br>- No
	Report or record shows nosocomial infections reported over the past 6 months	- Yes, observed	- Reported not seen<br>- No
	Written policies and procedures for identifying and managing drug-use problems	- Yes, observed	- Reported not seen<br>- No
	Surgery related mortality and morbidity review meetings at least monthly	- At least weekly<br>- At least monthly	- At least quarterly<br>- No specific timing<br>- None conducted
	Surgery related mortality and morbidity review meeting results recorded	- Yes, observed	- Reported not seen<br>- No
	Written guidelines or instructions for reporting on adverse events related to surgery	- Yes, observed	- Reported not seen<br>- No<br>- Don't know
	Notes or reports that show evidence of review and plan of action for the surgery reports	- Yes, documentation observed	- Yes, documentation reported not seen<br>- No
	Guidelines of postoperative infection definition	- Yes, documentation observed	- Yes, documentation reported not seen<br>- No
	Notes or reports that show evidence of review and plan of action for the reports about postoperative infection	- Yes, documentation observed	- Yes, documentation reported not seen<br>- No
	Indicator on deaths prior to discharge among patients who had a procedure in a surgical theater monitored	- Yes	- No
	Indicator on deaths prior to discharge among < 15 year old patients who had a procedure in a surgical theater monitored	- Yes	- No
	Indicator on post-operative surgical wound infection numbers monitored	- Yes	- No
	Facility monitor unplanned and unexpected hospital readmissions for any conditions	- Yes	- No
**Surgery equipment and medication (8)**	Basic operating table functioning	- Yes	- No
	Overhead operating light functioning	- Yes	- No
	Cardiac monitor functioning	- Yes	- No

For peer review only

			- Don't know
	EKG electrodes functioning	- Yes	- No<br>- Don't know
	Thermometer functioning	- Yes	- No<br>- Don't know
	Blood pressure apparatus functioning	- Yes	- No
	Suction apparatus functioning	- Yes	- No
	Anaesthesia machine available	- Yes available now	- No
**Surgery service (12)**	Wound debridement service available	- Yes, outpatient<br>- Yes, inpatient<br>- Yes both out and inpatient	- No
	Acute burn management service available	- Yes, outpatient<br>- Yes, inpatient<br>- Yes both out and inpatient	- No
	Closed repair of fracture service available	- Yes, outpatient<br>- Yes, inpatient<br>- Yes both out and inpatient	- No
	Closed reduction of dislocated joint service available	- Yes, outpatient<br>- Yes, inpatient<br>- Yes both out and inpatient	- No
	Cricothyroidotomy service available	- Yes, outpatient<br>- Yes, inpatient<br>- Yes both out and inpatient	- No
	Male circumcision service available	- Yes, outpatient<br>- Yes, inpatient<br>- Yes both out and inpatient	- No
	Hydrocele reduction service available	- Yes, outpatient<br>- Yes, inpatient<br>- Yes both out and inpatient	- No
	Biopsy of lymph node or mass service available	- Yes, outpatient<br>- Yes, inpatient<br>- Yes both out and inpatient	- No
	Appendectomy service available	- Yes, outpatient<br>- Yes, inpatient<br>- Yes both out and inpatient	- No
	Hernia repair service available	- Yes, outpatient<br>- Yes, inpatient<br>- Yes both out and inpatient	- No
	Open reduction and fixation service available	- Yes, outpatient<br>- Yes, inpatient<br>- Yes both out and inpatient	- No
	Any procedures using laparotomy service available	- Yes, outpatient<br>- Yes, inpatient<br>- Yes both out and inpatient	- No
**Internal medicine equipment and medication (3)**	Defibrillator available	- Yes, available now	- No
	Renal dialysis/haemodialysis machine available	- Yes, available now	- Yes, not available now<br>- No
	Lumbar puncture kit available	- Observed, at least one not expired	- Observed, available but expired<br>- Not observed, reported available but not seen<br>- Not observed, not available today<br>- Not observed, never available
**Internal medicine service (10)**	ART prescription and follow-up for U5 children service available	- Yes, ART prescription and clinical follow-up	- Yes, ART prescription, no clinical follow-up<br>- Yes, clinical follow-up, no ART prescription
	HIV/AIDS care and support service available	- Yes	- No
	Diagnose and/or manage diabetes available	- Yes	- No
	Diagnose and/or manage acute myocardial infraction available	- Diagnose, treat and patient follow-up	- Patient follow-up only<br>- No service/refer suspect case
	Diagnose and/or manage chronic respiratory disease available	- Yes	- No
	Diagnose and/or manage chronic kidney disease available	- Yes	- No
	Screening, diagnosis and/or treatment services for breast cancer available	- Yes	- No

For peer review only

	Screening, diagnosis and/or treatment services for colorectal cancer available	- Yes	- No
	Screening, diagnosis and/or treatment services for prostate cancer available	- Yes	- No
	Palliative care service available	- Yes	- No
**Paediatrics equipment and medication (8)**	Phototherapy machine available	- Yes, available now	- No
	Infant incubator available at outpatient	- Yes, available now	- Yes, not available now<br>- No
	Bed or location for KMC caregiver overnight	- Yes, observed	- Reported, not seen<br>- No
	Exchange transfusion blood available today	- Yes	- No
	Incubator available today	- Yes	- No
	Radiant warmer available today	- Yes	- No
	Artificial ventilation available today	- Yes	- No
	Device for intraosseous access available	- Observed, at least one not expired	- Observed, available but expired<br>- Not observed, reported available but not seen<br>- Not observed, not available today<br>- Not observed, never available
**Paediatrics service (3)**	KMC for premature or LBW used	- Yes	- No
	Newborn sepsis service available	- Yes	- No
	Newborn sepsis full antibiotics regimen and follow-up always available	- Yes always	- Yes sometimes<br>- Never
**OBGYN equipment and medication (10)**	Blood pressure apparatus functioning	- Yes	- No
	Infant scale (with 100 gram gradation) functioning	- Yes	- Don't know
	Resuscitation table (with heat source) functioning	- Yes	- No
	Self-inflating bag and mask for resuscitation functioning	- Yes	- No
	Newborn bag and mask size 0 for resuscitation of pre-term babies functioning	- Yes	- No
	Newborn bag and mask size 1 for resuscitation of term babies functioning	- Yes	- No
	Magnesium sulphate injection available	- Observed, at least one not expired	- Not observed, not available today
	Dexamethasone injection available	- Observed, at least one not expired	- Not observed, not available today<br>- Not observed, never available
	Misoprostol tablet 200 mcg available	- Observed, at least one not expired	- Not observed, not available today<br>- Not observed, never available
	Oxytocin injection available	- Observed, at least one not expired	- Not observed, not available today<br>- Not observed, never available
**OBGYN service (10)**	Active management of third stage labour (AMTSL) service available	- Yes	- No
	Monitoring and management of labor using a partograph service available	- Yes	- No
	Hygienic cord care service available	- Yes	- No
	Parenteral administration of antibiotics carried out	- Yes	- No
	Parenteral administration of oxytocic for treatment of postpartum haemorrhage carried out	- Yes	- No
	Assisted vaginal delivery using manual vacuum extraction (MVE) or forceps carried out	- Yes	- No, service not offered<br>- No, trained staff but no cases
	Manual removal of placenta carried out	- Yes	- No
	Removal of retained products of conception using D&C or manual vacuum aspiration carried out	- Yes	- No, service not offered
	PMTCT available	- Yes	- No
	Treatment of pre-invasive cervical cancer available	- Yes	- No
**Mental health / neurology (6)**	Any mental/neurological services offered	- Yes, inpatient only	- Not offered
	Mental health inpatient ward available	- Yes, inpatient only	- Not offered

For peer review only

	Neurological health inpatient ward available	- Yes, inpatient only	- Not offered
	Depression diagnosis and follow-up available	- Diagnoses and provides patient follow-up	- Diagnoses only<br>- Provides patient follow-up only<br>- No
	Psychosis diagnosis and follow-up available	- Diagnoses and provides patient follow-up	- Diagnoses only<br>- Provides patient follow-up only<br>- No
	Epilepsy diagnosis and follow-up available	- Diagnoses and provides patient follow-up	- Diagnoses only<br>- Provides patient follow-up only<br>- No

For peer review only

For peer review only

Additional file 2. All indicator heatmap by hospital bed number

	Level 4 small hospitals (n=23)	Level 4 large hospitals (n=23)	Level 5 & 6 hospitals (n=15)	Total (n=61)
**Human resources for health**				
At least five medical specialists	39%	44%	67%	48%
At least one surgeon	96%	96%	100%	97%
At least one internist	48%	57%	73%	57%
At least one paediatrician	70%	91%	100%	85%
At least one obstetrician-gynaecologist	87%	91%	100%	92%
At least one anaesthesiologist	44%	26%	93%	49%
**Lab tests**				
Rapid test				
HIV rapid test	87%	91%	87%	89%
Syphilis rapid test	96%	91%	100%	95%
Urine rapid test for pregnancy	100%	91%	100%	97%
Urine dipstick 3	35%	44%	53%	43%
Haemoglobin A1c rapid test	57%	57%	87%	64%
Colorimeter or haemoglobinometer	70%	61%	67%	66%
Glucometer	96%	96%	93%	95%
Glucometer test strips/discs	96%	100%	93%	97%
Basic lab test				
Any blood chemistry test	83%	78%	93%	84%
Renal function test	70%	74%	93%	77%
Other tests for bilirubin	65%	70%	67%	67%
ABO blood grouping testing	83%	74%	80%	79%
Rhesus blood grouping testing	83%	74%	87%	80%
Cross-match testing	57%	70%	80%	67%
Infectious disease test				
Onsite PCR for HIV viral load or HIV early-infant diagnosis	0%	4%	27%	8%
Xpert MTB/RIF rapid diagnostic testing for TB	61%	83%	87%	75%
Urine microscopy	91%	83%	93%	89%
Malaria smears	91%	83%	93%	89%
Microscopy test CSF body fluid counts	83%	78%	93%	84%
Cryptococcal antigen testing	74%	78%	80%	77%
Gram stain testing	91%	78%	93%	87%
Culture and sensitivity test	57%	74%	87%	71%
Blood cultures test	17%	22%	80%	34%
Advanced lab test				
Fine needle aspiration cytology	22%	44%	67%	41%
Core needle biopsy of lump specimen	35%	44%	67%	46%
Prostate Specific Antigen (PSA) Testing	48%	61%	80%	61%
Blood gas measurement	9%	13%	60%	23%
Cardiac marker (CK, Troponin) test	9%	30%	53%	28%
PAP smear read in facility	17%	13%	33%	20%
VIA/VILLI read in facility	96%	78%	73%	84%

For peer review only

Prepare and examine tissues or samples for cancer	0%	4%	53%	15%
**Oxygen and respiratory support**				
Surgery oxygen equipment functioning - Oxygen tank/cylinder with attached pressure gauge, pressure regulator	87%	83%	73%	82%
Maternity paediatric-sized oxygen delivery apparatus functioning	87%	74%	93%	84%
Outpatient pulse oximeter functioning	39%	48%	60%	48%
Maternity pulse oximeter functioning	35%	52%	67%	49%
Paediatrics/neonatal oxygen functioning	100%	100%	100%	100%
Emergency pulse oximeter functioning	65%	65%	100%	74%
Ventilator available	52%	52%	87%	61%
Chest tubes available	30%	61%	80%	54%
Adult CPAP equipment available	13%	30%	40%	26%
Emergency paediatrics and neonatal CPAP available	17%	26%	20%	21%
**Equipment**				
ECG offered	39%	35%	93%	51%
ECG equipment functioning	26%	35%	87%	44%
Ultrasound offered	83%	91%	100%	90%
Ultrasound equipment functioning	83%	91%	100%	90%
X-ray offered	87%	87%	100%	90%
X-ray equipment functioning	87%	83%	100%	89%
CT scan offered	26%	22%	80%	38%
CT scan equipment functioning	26%	17%	73%	34%
**24 7 availability**				
Emergency service 24/7	26%	13%	47%	26%
Medical officer onsite or oncall in facility 24/7	78%	70%	93%	79%
Formal triage tool used 24/7	26%	17%	47%	28%
Emergency surgery and anaesthesia 24/7	17%	13%	40%	21%
Emergency radiology 24/7	91%	87%	100%	92%
Emergency diagnostics 24/7	96%	87%	93%	92%
Emergency pharmacy 24/7	87%	83%	93%	87%
Blood transfusion	91%	83%	100%	90%
**Infection prevention and control**				
Facility has IPC guideline	87%	70%	87%	80%
Technical IPC committee	96%	100%	93%	97%
Dedicated (full-time) IPC staff	57%	52%	73%	59%
Guidelines for cleaning	48%	57%	33%	48%
Step-by-step techniques for specific tasks	44%	57%	60%	53%
Cleaning roster or schedule specifying responsibility	48%	61%	73%	59%
Cleaning beds observed	44%	61%	67%	56%
Cleaning counters/tables observed	48%	61%	60%	56%
Cleaning toilets observed	48%	70%	67%	61%
**Quality and safety**				
Monthly quality assurance committee meeting	26%	35%	53%	36%
Documentation of quality assurance information reviewed	52%	44%	67%	53%
Ongoing quality improvement using Plan-Do-Check-Study-Act approach	64%	67%	67%	66%
Multi-disciplinary quality improvement team	65%	61%	80%	67%
Departmental specific work improvement teams	65%	61%	80%	67%

For peer review only

Documentation of improvement achieved as a result of quality improvement process	48%	52%	33%	46%
Kenya Quality Model for Health completed in 2017 or 2018	48%	44%	60%	49%
Formal case reviews carried out at least monthly	57%	57%	67%	59%
Death reviews results recorded	91%	91%	100%	93%
Guidelines of events that are considered adverse and required to be reported	22%	35%	33%	30%
Notes or reports that show evidence of review and plan of action for the reports about adverse events	22%	39%	27%	30%
Written guidelines that define nosocomial infections and the process for reporting	9%	9%	40%	16%
Report or record shows nosocomial infections reported over the past 6 months	9%	4%	33%	13%
Written policies and procedures for identifying and managing drug-use problems	70%	65%	67%	67%
Surgery related mortality and morbidity review meetings at least monthly	44%	39%	60%	46%
Surgery related mortality and morbidity review meeting results recorded	44%	57%	53%	51%
Written guidelines or instructions for reporting on adverse events related to surgery	9%	26%	33%	21%
Notes or reports that show evidence of review and plan of action for the surgery reports	9%	17%	27%	16%
Guidelines of postoperative infection definition	17%	26%	33%	25%
Notes or reports that show evidence of review and plan of action for the reports about postoperative infection	13%	17%	13%	15%
Indicator on deaths prior to discharge among patients who had a procedure in a surgical theater monitored	9%	22%	53%	25%
Indicator on deaths prior to discharge among < 15 year old patients who had a procedure in a surgical theater monitored	9%	17%	47%	21%
Indicator on post-operative surgical wound infection numbers monitored	9%	17%	40%	20%
Facility monitor unplanned and unexpected hospital readmissions for any conditions	30%	26%	40%	31%
**Surgery**				
Surgery equipment & medication				
Basic operating table functioning	96%	96%	100%	97%
Overhead operating light functioning	96%	96%	100%	97%
Cardiac monitor functioning	87%	83%	100%	89%
EKG electrodes functioning	83%	70%	100%	82%
Thermometer functioning	96%	87%	93%	92%
Blood pressure apparatus functioning	70%	91%	93%	84%
Suction apparatus functioning	100%	96%	100%	98%
Anaesthesia machine available	83%	74%	93%	82%
Surgery service				
Wound debridement service available	100%	100%	100%	100%
Acute burn management service available	96%	96%	100%	97%
Closed repair of fracture service available	96%	91%	100%	95%
Closed reduction of dislocated joint service available	96%	91%	100%	95%
Cricothyroidotomy service available	78%	57%	80%	71%
Male circumcision service available	100%	96%	93%	97%
Hydrocele reduction service available	100%	91%	93%	95%
Biopsy of lymph node or mass service available	100%	100%	100%	100%
Appendectomy service available	100%	96%	100%	98%
Hernia repair service available	100%	96%	100%	98%
Open reduction and fixation service available	100%	83%	100%	93%
Any procedures using laparotomy service available	100%	96%	100%	98%

For peer review only - http://bmjopen.bmj.com/site/about/guidelines.xhtml

**Internal medicine**				
Internal medicine equipment & medication				
Defibrillator available	57%	39%	73%	54%
Renal dialysis/haemodialysis machine available	44%	65%	93%	64%
Lumbar puncture kit available	22%	26%	80%	38%
Internal medicine service				
ART prescription and follow-up for U5 children service available	96%	96%	93%	95%
HIV/AIDS care and support service available	100%	96%	100%	98%
Diagnose and/or manage diabetes available	100%	100%	100%	100%
Diagnose and/or manage acute myocardial infraction available	83%	74%	93%	82%
Diagnose and/or manage chronic respiratory disease available	100%	100%	100%	100%
Diagnose and/or manage chronic kidney disease available	83%	91%	100%	90%
Screening, diagnosis and/or treatment services for breast cancer available	78%	87%	93%	85%
Screening, diagnosis and/or treatment services for colorectal cancer available	35%	30%	60%	39%
Screening, diagnosis and/or treatment services for prostate cancer available	61%	83%	87%	75%
Palliative care service available	48%	65%	100%	67%
**Paediatrics**				
Paediatrics equipment & medication				
Phototherapy machine available	52%	52%	67%	56%
Infant incubator available	74%	78%	87%	79%
Bed or location for KMC caregiver overnight	61%	70%	80%	69%
Exchange transfusion blood available today	26%	17%	60%	31%
Incubator available today	96%	100%	100%	98%
Radiant warmer available today	91%	96%	93%	93%
Artificial ventilation available today	48%	57%	73%	57%
Device for intraosseous access available	17%	30%	53%	31%
Paediatrics service				
KMC for premature or LBW used	83%	87%	93%	87%
Newborn sepsis service available	100%	96%	100%	98%
Newborn sepsis full antibiotics regimen and follow-up always available	91%	87%	87%	89%
**OBGYN**				
OBGYN equipment & medication				
Blood pressure apparatus functioning	91%	100%	100%	97%
Infant scale (with 100 gram gradation) functioning	87%	96%	100%	93%
Resuscitation table (with heat source) functioning	91%	100%	93%	95%
Self-inflating bag and mask for resuscitation functioning	87%	91%	80%	87%
Newborn bag and mask size 0 for resuscitation of pre-term babies functioning	91%	91%	87%	90%
Newborn bag and mask size 1 for resuscitation of term babies functioning	96%	96%	93%	95%
Magnesium sulphate injection available	96%	96%	100%	97%
Dexamethasone injection available	91%	91%	73%	87%
Misoprostol tablet 200 mcg available	74%	70%	73%	72%
Oxytocin injection available	100%	96%	100%	98%
OBGYN service				
Active management of third stage labour (AMTSL) service available	100%	100%	100%	100%
Monitoring and management of labor using a partograph service available	96%	96%	100%	97%

For peer review only

Hygienic cord care service available	100%	91%	93%	95%
Parenteral administration of antibiotics carried out	100%	100%	100%	100%
Parenteral administration of oxytocic for treatment of postpartum haemorrhage carried out	100%	100%	100%	100%
Assisted vaginal delivery using manual vacuum extraction (MVE) or forceps carried out	57%	74%	73%	67%
Manual removal of placenta carried out	100%	100%	100%	100%
Removal of retained products of conception using D&C or manual vacuum aspiration carried out	83%	83%	100%	87%
PMTCT available	100%	100%	100%	100%
Treatment of pre-invasive cervical cancer available	44%	48%	53%	48%
**Mental health / neurology**				
Any mental/neurological services offered	65%	96%	93%	84%
Mental health inpatient ward available	26%	48%	80%	48%
Neurological health inpatient ward available	13%	22%	67%	30%
Depression diagnosis and follow-up available	48%	87%	87%	72%
Psychosis diagnosis and follow-up available	52%	91%	87%	75%
Epilepsy diagnosis and follow-up available	57%	91%	87%	77%

For peer review only

Additional file 3. Comparing internship hospitals sampled in KHHFA and others

	61 KHHFA hospitals	12 other hospitals
Level		
- Level 4	44	11
- Level 5	15	1
- Level 6	2	0
Ownership		
- MoH	54	0
- Private	2	4
- FBO	5	8
Bed number		
- Mean (SD)	262.9 (213.4)	171.2 (71.0)
- Median (IQR)	200 (156, 276)	180 (106, 221)
Total delivery		
- Mean	4585.1 (2747.0)	1349.4 (945.7)
- Median (IQR)	3980.7 (2972.3, 5502.7)	1150.4 (649.7, 1683.5)
Live births		
- Mean	4466.9 (2691.9)	1321.3 (927.8)
- Median (IQR)	3814.3 (2844, 5384.3)	1138.4 (626.5, 1630)
C-section		
- Mean	1190.3 (917.6)	536.1 (270.7)
- Median (IQR)	987.3 (734.3, 1319.3)	536.6 (289, 708.5)

For peer review only

STROBE Statement—Checklist of items that should be included in reports of ***cross-sectional studies***

	Line No	Recommendation
**Title and abstract**	1-3	(*a*) Indicate the study's design with a commonly used term in the title or the abstract
		(*b*) Provide in the abstract an informative and balanced summary of what was done and what was found
**Introduction**		
Background/rationale	82-127	Explain the scientific background and rationale for the investigation being reported
Objectives	121-127	State specific objectives, including any prespecified hypotheses
**Methods**		
Study design	131-132	Present key elements of study design early in the paper
Setting	129-144	Describe the setting, locations, and relevant dates, including periods of recruitment, exposure, follow-up, and data collection
Participants	129-144	(*a*) Give the eligibility criteria, and the sources and methods of selection of participants
Variables	146-170	Clearly define all outcomes, exposures, predictors, potential confounders, and effect modifiers. Give diagnostic criteria, if applicable
Data sources/ measurement	129-144	For each variable of interest, give sources of data and details of methods of assessment (measurement). Describe comparability of assessment methods if there is more than one group
Bias	161-170	Describe any efforts to address potential sources of bias
Study size	131-136	Explain how the study size was arrived at
Quantitative variables	146-170	Explain how quantitative variables were handled in the analyses. If applicable, describe which groupings were chosen and why
Statistical methods	173-187	(*a*) Describe all statistical methods, including those used to control for confounding
		(*b*) Describe any methods used to examine subgroups and interactions
		(*c*) Explain how missing data were addressed
		(*d*) If applicable, describe analytical methods taking account of sampling strategy
		(*e*) Describe any sensitivity analyses
**Results**		
Participants	194-204	(a) Report numbers of individuals at each stage of study—eg numbers potentially eligible, examined for eligibility, confirmed eligible, included in the study, completing follow-up, and analysed
		(b) Give reasons for non-participation at each stage
		(c) Consider use of a flow diagram
Descriptive data	194-204	(a) Give characteristics of study participants (eg demographic, clinical, social) and information on exposures and potential confounders
		(b) Indicate number of participants with missing data for each variable of interest
Outcome data	206-304	Report numbers of outcome events or summary measures
Main results	206-304	(*a*) Give unadjusted estimates and, if applicable, confounder-adjusted estimates and their precision (eg, 95% confidence interval). Make clear which confounders were

For peer review only - http://bmjopen.bmj.com/site/about/guidelines.xhtml

		adjusted for and why they were included
		(*b*) Report category boundaries when continuous variables were categorized
		(*c*) If relevant, consider translating estimates of relative risk into absolute risk for a meaningful time period
Other analyses	206-304	Report other analyses done—eg analyses of subgroups and interactions, and sensitivity analyses
**Discussion**		
Key results	307-314	Summarise key results with reference to study objectives
Limitations	376-420	Discuss limitations of the study, taking into account sources of potential bias or imprecision. Discuss both direction and magnitude of any potential bias
Interpretation	316-394	Give a cautious overall interpretation of results considering objectives, limitations, multiplicity of analyses, results from similar studies, and other relevant evidence
Generalisability	382-394	Discuss the generalisability (external validity) of the study results
**Other information**		
Funding	451-453	Give the source of funding and the role of the funders for the present study and, if applicable, for the original study on which the present article is based

*Give information separately for exposed and unexposed groups.

Note: An Explanation and Elaboration article discusses each checklist item and gives methodological background and published examples of transparent reporting. The STROBE checklist is best used in conjunction with this article (freely available on the Web sites of PLoS Medicine at http://www.plosmedicine.org/, Annals of Internal Medicine at http://www.annals.org/, and Epidemiology at http://www.epidem.com/). Information on the STROBE Initiative is available at www.strobe-statement.org.

For peer review only

For peer review only - http://bmjopen.bmj.com/site/about/guidelines.xhtml

BMJ Open

Characterising Kenyan hospitals' suitability for medical officer internship training: secondary data analysis of a cross-sectional study

Journal:	*BMJ Open*
Manuscript ID	bmjopen-2021-056426.R1
Article Type:	Original research
Date Submitted by the Author:	14-Feb-2022
Complete List of Authors:	Zhao, Yingxi; University of Oxford, Nuffield Department of Medicine<br>Osano, Boniface; Kenya Paediatric Research Consortium<br>Were, Fred; Kenya Paediatric Research Consortium<br>Kiarie, Helen; Ministry of Health<br>Nicodemo, Catia; University of Oxford, CHSEO<br>Gathara, David; Kenya Medical Research Institute, Wellcome Trust Research Program<br>English, Mike; University of Oxford, Nuffield Department of Medicine ; KEMRI-Wellcome Trust Research Programme Nairobi,
**Primary Subject Heading**:	Medical education and training
Secondary Subject Heading:	Health services research, Medical education and training, Global health
Keywords:	Organisation of health services < HEALTH SERVICES ADMINISTRATION & MANAGEMENT, Quality in health care < HEALTH SERVICES ADMINISTRATION & MANAGEMENT, Health & safety < HEALTH SERVICES ADMINISTRATION & MANAGEMENT, MEDICAL EDUCATION & TRAINING

SCHOLARONE™
Manuscripts

I, the Submitting Author has the right to grant and does grant on behalf of all authors of the Work (as defined in the below author licence), an exclusive licence and/or a non-exclusive licence for contributions from authors who are: i) UK Crown employees; ii) where BMJ has agreed a CC-BY licence shall apply, and/or iii) in accordance with the terms applicable for US Federal Government officers or employees acting as part of their official duties; on a worldwide, perpetual, irrevocable, royalty-free basis to BMJ Publishing Group Ltd ("BMJ") its licensees and where the relevant Journal is co-owned by BMJ to the co-owners of the Journal, to publish the Work in this journal and any other BMJ products and to exploit all rights, as set out in our licence.

The Submitting Author accepts and understands that any supply made under these terms is made by BMJ to the Submitting Author unless you are acting as an employee on behalf of your employer or a postgraduate student of an affiliated institution which is paying any applicable article publishing charge ("APC") for Open Access articles. Where the Submitting Author wishes to make the Work available on an Open Access basis (and intends to pay the relevant APC), the terms of reuse of such Open Access shall be governed by a Creative Commons licence – details of these licences and which Creative Commons licence will apply to this Work are set out in our licence referred to above.

Other than as permitted in any relevant BMJ Author's Self Archiving Policies, I confirm this Work has not been accepted for publication elsewhere, is not being considered for publication elsewhere and does not duplicate material already published. I confirm all authors consent to publication of this Work and authorise the granting of this licence.

For peer review only

Characterising Kenyan hospitals' suitability for medical officer internship training: a secondary data analysis of a cross-sectional study

Author: Yingxi Zhao[1], Boniface Osano[2,3], Fred Were[2,3], Helen Kiarie[4], Catia Nicodemo[5], David Gathara[6,7], Mike English[1,6]

Affiliation:

1. Oxford Centre for Global Health Research, Nuffield Department of Medicine, University of Oxford, Oxford, UK
2. University of Nairobi Department of Paediatrics and Child Health, Nairobi, Kenya
3. Kenya Paediatric Research Consortium (KEPRECON), Nairobi, Kenya
4. Division of Monitoring & Evaluation, Ministry of Health, Nairobi, Kenya
5. Nuffield Department of Primary Care Health Sciences, University of Oxford, Oxford, UK
6. KEMRI-Wellcome Trust Research Programme, Nairobi, Kenya
7. MARCH Centre, London School of Hygiene and Tropical Medicine, London, UK

For peer review only

Corresponding: Yingxi Zhao
+44 (0) 7491422427
yingxi.zhao@ndm.ox.ac.uk
Oxford Centre for Global Health Research
Nuffield Department of Medicine
University of Oxford
S Parks Rd, Oxford OX1 3SY, UK

Version: 4.1 Updated Feb 14, 2022

Word count: 4,080

For peer review only

For peer review only - http://bmjopen.bmj.com/site/about/guidelines.xhtml

Abstract

Objective: To characterise the capacity of Kenya internship hospitals to understand whether they are suitable to provide internship training for medical doctors.

Design: A secondary data analysis of a cross-sectional health facility assessment (Kenya Harmonized Health Facility Assessment 2018).

Setting and population: We analyzed 61 out of all 74 Kenyan hospitals that provide internship training for medical doctors.

Outcome measures: Comparing against the minimum requirement outlined in the national guidelines for medical officer interns, we filtered and identified 166 indicators from the KHFA survey questionnaire and grouped them into 12 domains. An overall capacity index was calculated as the mean of 12 domain-specific scores for each facility.

Results: The average overall capacity index is 69% (95%CI 66%-72%) for all internship training centres. Hospitals have moderate capacity (over 60%) for most of the general domains, though there is huge variation between hospitals and only 29 out of 61 hospitals have five or more specialists assigned, employed, seconded or part-time - as required by the national guideline. Quality and safety score was low across all hospitals with an average score of 40%. As for major specialties, all hospitals have good capacity for surgery and obstetrics-gynaecology, while mental health was poorest in comparison. Level 5 & 6 facilities (provincial and national hospitals) have higher capacity scores in all domains when compared with Level 4 hospitals (equivalent to district hospitals).

Conclusion: Major gaps exist in staffing, equipment and service availability of Kenya internship hospitals. Level 4 hospitals (equivalent to district hospitals) are more likely to have a lower capacity index, leading to low quality of care, and should be reviewed and

For peer review only

improved to provide appropriate and well-resourced training for interns and to utilise appropriate resources to avoid improvising.

Keywords

Internship, hospital, capacity, service availability, human resources for health, quality and safety

Strengths and limitations of this study:

- Using data collected from a national health facility assessment, our study is able to characterise the capacity of 61 out of 74 Kenya internship hospitals and contrast findings across levels of hospitals and with the minimum requirements outlined in the national guidelines for medical officer internship training.
- Our analyses have clear implications for Kenya policy-makers to improve the conditions of internship hospitals. More widely, our findings point to the need to carefully consider the potential consequences of rapidly expanding medical training and of appropriate planning and financing for new internship centres, especially in rural areas.
- Our analysis was only limited to the 61 internship training hospitals sampled by KHFA, mostly public hospitals; and KHFA data was collected n 2018 prior to Covid-19 therefore it is possible that the capacity of Kenyan internship training centres has improved since 2018.
- To assess the capacity we selected 166 signal indicators from the 3000 questions from KHFA survey, however the indicators selected focused only on the structural

For peer review only

80 and organizational features of internship training hospitals, and the selection process

81 could be somewhat subjective.

For peer review only

For peer review only - http://bmjopen.bmj.com/site/about/guidelines.xhtml

Background

In Kenya as in many countries, doctors are first trained for 5 or 6 years in medical school (typically 2 or 3 years pre-clinical, 3 years clinical). This is followed by a mandatory one-year internship prior to receiving a license to practice from the national regulatory board, i.e. the Kenya Medical Practitioners and Dentists Council, overseen by the National Ministry of Health [1]. The Kenyan medical internship included supervised rotation in 4 major departments (surgery, internal medicine, paediatrics and child health, and obstetrics and gynaecology [OBGYN]) in one Internship training centre but since 2020 mental health and community health practice became added requirements. The competencies medical interns are expected to develop e.g. compulsory procedures that should be carried out either independently or under supervision, are outlined in national guidelines and in greater detail in interns' personal log books [2]. After successfully completing internship and subsequent licensure individuals can practice medicine unsupervised as general medical officers in different types of facilities.

Medical internships are undertaken in specific hospitals approved for this purpose after evaluation by the Kenya Medical Practitioners and Dentists Council, and all hospitals should meet and maintain their minimum requirements. These sites are an essential component of the health and education system producing a country's future physicians [3]. Ensuring internship training hospitals are well-staffed and equipped is important so that medical graduates can be supervised and supported to consolidate their knowledge and skills, including of common clinical procedures, and become competent medical doctors [4].

For peer review only - http://bmjopen.bmj.com/site/about/guidelines.xhtml

Improving internship training could also improve the quality of care and patient safety as interns are at the frontline of patient management especially in LMICs [5].

As with other African countries, medical schools in Kenya are expanding to meet human resources for health gaps. The number of Kenyan medical schools has increased dramatically from 2 in 2004 to 12 in 2018 [6], meanwhile the number of medical doctors graduating almost doubled from 287 in 2006 to 573 in 2018 [7,8]. The number of internship training centres recognised by the Ministry of Health and regulators also increased from 6 to 74 over the past 15 years and includes private or mission/faith-based hospitals. [9,10]. Many new training centres that were formerly district hospitals but are now county hospitals in Kenya (at Level 4 of its 6-level health system, usually primary facilities including district hospitals) became recognised as internship centres alongside more established secondary or tertiary regional and national hospitals (at Level 5 and 6 respectively). However, these county hospitals may have shortages of staff, limited diagnostic facilities, and frequent stockout of medications and diagnostics reagents. Such lack of organizational structures and resources, i.e. inadequate capacity, would mean these facilities are not ready and suitable for providing internship training, for example a qualitative study in Kenya suggested that these county hospitals are “not organized for training purposes” [9].

Previous studies conducted in Kenya between 2012 and 2014 highlighted poor working conditions and experiences for interns at district hospitals, however they were limited in scale (all including less than 10 hospitals) and did not compare hospitals at different levels and sizes [9,11,12]. Using data collected from the Kenya Harmonized Health Facility Assessment 2018 (KHFA), we address the question ‘what capacity do facilities offering

For peer review only - http://bmjopen.bmj.com/site/about/guidelines.xhtml

internship training have?' To do this we characterise the capacity of 61 internship hospitals contrasting findings across levels of hospitals and with the minimum requirements outlined in the national guidelines for medical officer internship training [2], with a specific focus on the 'structural' component of the Donabedian quality of care framework (structure, process and outcome) [13].

Methods

Data sources

This is a secondary data analysis using data from the 2018 Kenya Harmonized Health Facility Assessment (KHFA). Details of the KHFA and its methodology are publicly available [14]. KHFA used the Kenya Health Master Facility List (MFL) as the sampling frame, randomly sampled 2,980 out of 10,535 health facilities in Kenya, but purposely included all secondary hospitals, public primary hospitals and maternity and nursing homes. Sixty one out of seventy four internship training centres were sampled in KHFA. The survey included over 3000 questions covering five main modules on resource availability, management and finance, readiness, quality and safety of health care, and data verification (information systems). Data were collected through facility audit, observation and validation, provider interview and record review. To supplement the KHFA data we extracted: the inpatient bed number in 2020 from the MFL [15]; the total delivery count between 2018 to 2020 from the national Kenya Health Information System (previously District Health Information Software-2 [DHIS2]); and verified key human resources for health data for six hospitals with incomplete information through direct contact with hospitals.

For peer review only - http://bmjopen.bmj.com/site/about/guidelines.xhtml
For peer review only

Domains and indicators

We focus on the ‘structure’ component of the Donabedian quality of care framework to characterise hospitals. Specifically, we explore staffing, equipment and service availability and whether they fulfil the minimum requirement outlined in the Kenyan National Guidelines for Internship Training of Medical and Dental Officer Interns (2019) and the National Guidelines and Log Book for Medical Officer Interns (2019) [2]. For example, the guidelines require that each internship training centre: i) has a minimum of 5 medical specialists, covering paediatrics & child health, general surgery, internal medicine, obstetrics-gynaecology (OBGYN) and family medicine, ii) is fully operational on a 24-hour basis including accident & emergency, diagnostic and pharmacy services, iii) enables interns to observe and/or perform certain procedures to fulfill competency for each specialty. Examples of the latter in surgery include a total of 36 competency procedures such as appendicectomy or repair of inguinal hernias. This requires that these services be provided by internship training centres [2].

Two authors (ME, YZ) initially filtered out 277 indicators from the 3000 questions from KHFA to identify those most relevant to the minimum criteria defined by the national internship guidelines. Two authors (BO, FW) who are medical school faculty and who supervise medical officer interns subsequently reviewed these indicators identifying those felt to be most useful to characterizing hospitals as contexts for internship training by. A final list of 166 indicators was agreed by all four authors and selected for further analysis as “signal indicators” of the minimum requirements for internship training centres. All the indicators were converted into binary responses with 1 representing “met/available” and 0

For peer review only

For peer review only - http://bmjopen.bmj.com/site/about/guidelines.xhtml

representing “not met/unavailable”. We grouped indicators into 12 logical domains described in brief in Table 1 with further detail of all indicators available in online supplement material 1.

Analysis

For each domain, we calculated a score as a percentage based on the number of indicators with a score of 1 (met/available) divided by the total number of indicators (those met/available + those not met/unavailable). We generated an overall capacity index as the unweighted mean of all 12 domain scores for each facility. We stratified the 61 included internship training centres into three categories based on hospital administrative level and inpatient bed number. Kenya has a six service-level system based on the essential package for health, whereas Level 1 refers to community-level, Level 2 dispensaires and clinics, Level 3 health centres, Level 4 primary facilities including district hospitals, Level 5 secondary provicinal hospitals and Level 6 tertiary and national hospitals. We first grouped all internship hospitals that are Level 5 & 6 together (bed number 174-1455, n=15) as historically they have been better resourced; for the the internship hospitals that are level 4, we divided them based on bed number into: Level 4 small hospitals (bed number 82-175, n=23) and Level 4 large hospitals (bed number 176-320, n = 23). Data analysis was conducted using Stata version 16 (Stata Corporation, Texas, USA).

Ethics approval

The Kenya Ministry of Health, Division of Monitoring and Evaluation, sought approval for, planned and execute the 2018 Kenya Harmonized Health Facility Assessment. Written

For peer review only

informed consent was sought by in-charge or acting in-charge of all the facilities surveyed. Permission was then granted to use these data for the secondary analysis reported here with the purpose of improving quality of health service provision in Kenya.

Patient and public involvement

Patients and the public were not directly involved in the design, recruitment or conducting of this study.

Results

Sixty one out of seventy four (61/74) internship training centres are sampled in KHFA and are mostly public hospitals owned by the Ministry of Health (n=54). The 13/74 hospitals not sampled are either private hospitals (n=4), faith-based or mission hospitals (n=8) or a military hospital (n=1). Comparing hospital characteristics retrieved from DHIS2 and MFL between the 61 KHFA-sampled hospitals with 12 of the 13 other hospitals (the military hospital did not report to DHIS2 or MFL), the 61 KHFA hospitals are slightly larger in terms of hospital beds (median and inter-quarter range [IQR] 200 [156, 276] vs. 180 [106, 221]), and report conducting three times as many deliveries (median annual deliveries 3981 vs. 1150) and twice as many Caesarean sections (median 987 vs. 537 per year). Detailed characteristics comparing the 61 KHFA internship training centres and these 12 others are presented in online supplement material 2.

Overall capacity

For peer review only

The distribution of capacity score by domain is presented in Figure 1 and Table 2. Ideally, an internship centre should score 100% against the (minimum) signal indicators but the overall capacity index is 69% (95%CI 66%-72%) for all internship training centres, and 63%, 67% and 81% for Level 4 small, Level 4 large and Level 5 & 6 hospitals respectively. Hospitals have moderate capacity for most of the general domains other than quality and safety. Surgery and OBGYN have higher scores regardless of hospital size, level and location, suggesting that the capacity for these two specialties is better across all facilities. Level 5 & 6 facilities have higher capacity scores in all domains when compared with Level 4 hospitals.

Human resources

The 61 internship training hospitals had a median of 14 post-licensure medical officers, 4 medical specialists, 25 clinical officers, 210 nurses and midwives, 14 medical laboratory technologists, 5 radiographers and 14 allied health professionals. Differences between small and large Level 4 hospitals were small for most cadres, whereas Level 5 & 6 hospitals have, on average, double the numbers of specialists, nurses and midwives (Table 3). The national guideline specifically requires at least 5 medical specialists to act as the main clinical teachers and supervisors for medical officer interns. However, only 29 out of 61 internship training centres had at least five medical specialists assigned, employed or seconded (including part-time staff). Most hospitals had at least one surgeon (n=59/61), paediatrician (n=52/61) and an obstetrician-gynaecologist (n=56/61), whereas only 35/61 hospitals have at least an internal medicine specialist. Although not a requirement from the regulator, only 30/61 hospitals have a physician anaesthesiologist (Figure 2) with anaesthesia mostly provided by clinical officers (51/61 hospitals have at least one anaesthetist clinical officer).

For peer review only

The 4 well-established university teritary hospitals have better human resources and all but one fulfilled all the requirements.

Diagnostics and supportive care

As for laboratory tests, internship training centres overall have moderate availability (over 60%) for rapid tests and basic laboratory lab tests (Figure 3 and online supplement material 3), although ability to offer urine dipstick testing was present in fewer than 50% hospitals (n=26/61). 54/61 hospitals have rapid HIV tests, 16 hospitals provided onsite polymerase chain reaction (PCR) testing for HIV but only 5 had onsite HIV viral load testing. An additional 19 hospitals relied on off-site PCR testing. Availability for advanced lab tests is poor across all hospitals, only 12/61 hospitals can read papanicolaou test (PAP smear) results and prepare and examine any tissues or samples for cancer diagnosis onsite. Level 5 and 6 hospitals on average have better availability of diagnostics than Level 4 hospitals, especially for infectious disease and advanced lab tests.

Internship training hospitals vary when assessed for 10 oxygen and respiratory support indicators. While all hospitals were reported to have functional paediatric and neonatal oxygen, only 32 hospitals have ventilators available for adults and 13 hospitals had continuous positive airway pressure (CPAP) available for neonates (at the time of survey in 2018 pre-Covid-19). Lastly for other general equipment, around 55 hospitals reported functioning ultrasound and X-ray services. In comparison, functional electrocardiogram (ECG) (27/61) and computerised tomography (CT) scan (21/61) availability was poor in most facilities.

For peer review only

Service continuity and safety

The national guideline requires that internship hospitals need to be fully operational on a 24-hour basis including accident & emergency, laboratory diagnostic services, and pharmacy which was investigated under "24/7 availability". 48/61 hospitals reported having general medical officers or emergency medicine specialists onsite or on-call within facility. While emergency radiology, laboratory diagnostics (other than rapid test) and pharmacy services are available 24/7 in most facilities, only 13/61 hospitals reported availability of major emergency surgery and anesthesia 24/7, and only 16 hospitals provide 24-hour emergency services using a structured triage tool such as the WHO integrated triage tool.

The domain average score for infection prevention and control is 66%. Most internship training centres have infection prevention and control (IPC) guidelines and technical IPC committees, however, the actual procedures of cleaning beds, counters/tables, toilets were only done in around half of the hospitals on the day of KHFA survey observation. Twenty one input/structure indicators on quality and safety are also included in the current analysis. All but one indicator (death review results recorded) performed poorly across all facilities. Most facilities do not have observed guidelines for adverse event reporting, nosocomial infection reporting, surgical adverse event reporting or postoperative infection definitions. Level 5 & 6 hospitals do not necessarily perform better than Level 4 hospitals in this domain.

Major specialties

For the four major specialties we used data on both specialty-specific equipment & medicines and specialty service areas (online supplement material 3). For surgical and OBGYN scores, internship training centres in general have both functional equipment &

For peer review only - http://bmjopen.bmj.com/site/about/guidelines.xhtml

available medicine (median 100% for surgery, 100% for OBGYN) and also good service availability for procedures relevant to the national guideline and log book (median 100% for surgery, 90% for OBGYN). The only indicator that had a lower score is cricothyroidotomy, where only 43/61 hospitals provide such service; and misoprostol tablet availability, where 44/61 hospitals have this medication available.

For internal medicine, availability of equipment and medicine is low (median 67%) with huge variation across facilities. 33/61 hospitals have a defibrillator, 39/61 have a renal dialysis/hemodialysis machine and 23/61 hospitals have lumbar puncture kits – for the rest of the hospitals they will need to improvise to perform lumbar punctures or cannot provide such a service. Service availability for internal medicine is good on average (median 90%), however availability for more advanced conditions e.g. colorectal cancer screening, diagnosis and/or treatment, and palliative care is limited especially for Level 4 small hospitals. Similar with internal medicine, the availability of equipment for paediatrics is lower as compared with other specialties (median 63%). Only 19/61 hospitals had exchange blood transfusion capacity and similarly only 19/61 hospitals had devices for intraosseous (IO) access. The three indicators of paediatric service availability (kangaroo mother care, sepsis, antibiotics regimen and follow-up) had good availability.

For peer review only

Medical officer interns are also required to rotate in mental health for eight weeks during their one-year internship and are required to manage acute mental health emergencies and common mental health conditions. While 52/61 hospitals offer mental health and/or neurological services, only 17 hospitals have a physician psychiatrist. Sixteen hospitals have mental health inpatient wards, other hospitals either only provide outpatient services or

lack these services. Only half of Level 4 small hospitals offer depression, psychosis and epilepsy diagnosis and follow-up services.

Discussion

Our analysis on the capacity of Kenya internship training hospitals suggested that in 2018 the overall staffing, equipment and service availability was inadequate when compared with the regulator's requirements. Level 4/district, smaller hospitals (23 of the 61 assessed) were more likely to have fewer human resources, equipment and specialty services, and less likely to meet quality and safety indicators. The implication is that hospitals with lower capacity may either have to improvise to offer core services or refer patients to other facilities. This could significantly influence the quality of training as interns might not be able to fully consolidate their knowledge and skills.

Over half of the internship training hospitals did not have five or more specialists as required by the regulator, the medical council, which suggests inadequate training and supervision of medical officer interns. Internal medicine specialists and anaesthesiologists were most often lacking suggesting that in many centres non-specialist but licensed general medical or specialist clinical officers fill these roles. Even in these larger Kenyan hospitals such personnel will also likely therefore be leading provision of care for severe COVID-19. Where medical officers are themselves also scarce, medical officer interns may become the only “in-house” doctor on call and be forced to take on full responsibility for patient care potentially threatening patient safety if interns are forced to care for serious, acute medical and surgical conditions without appropriate supervision [9,16,17]. The likely challenges to

For peer review only

provision of high quality safe care are further suggested by poor scores of all but tertiary hospitals against organisational indicators of quality and safety practice. In fact guidelines or instruction for quality and safety were rarely present and there seemed very limited evidence that quality and safety indicators are monitored, reviewed, reported and acted upon. These findings are consistent with reports highlighting challenges with patient safety in low- and middle-income country (LMIC) hospitals [18].

Internship hospitals appear most able to provide appropriate essential resources and capacities in the major disciplines of surgery and OBGYN. Challenges were especially noted in the disciplines of internal medicine and paediatrics and neonatology. Many facilities lack ECG machines, CT scans, defibrillators, equipment used for peritoneal or hemo-dialysis, and lumbar puncture kits. More specific to neonatal and child health were lack of capacity in ability to provide exchange transfusion, neonatal CPAP and intraosseous access. Capacity to offer mental health and neurological care was especially low most particularly in Level 4 small hospitals, likely due to these only being listed as internship requirements in 2020, and that Kenya has a severe shortage of psychiatrists and neurologists as well as psychiatric nurses [19]. For facilities without capacity in the major disciplines now including mental health and neurology services it is likely that hospitals are forced to refer patients elsewhere or improvise approaches to care in order to serve patients. Interns' training experience is therefore likely to be very varied and more attention may therefore need to be paid to planning how interns can be provided with adequate training in all specialties if this is a key long-term aim.

For peer review only

For peer review only - http://bmjopen.bmj.com/site/about/guidelines.xhtml

Kenya has dramatically increased its medical training volume hoping to produce 9,000 new graduate medical physicians by 2030 to narrow its staffing gap [6]. More hospitals especially at county-level (district hospitals) are accredited as internship training centres. These hospitals are usually smaller in size and more likely to be distant from the well-established university tertiary hospitals in major cities that scored much higher across domians in our analysis. Rural rotations and residencies have been recommended by the WHO to increase health worker retention [20]. Students or interns in these settings may also be more likely to learn hands-on clinical procedures and be actively engaged in patient care [21] as opposed to tertiary hospitals where the presence of more specialists and general medical officers may result in interns undertaking more administrative work. However, the prerequisite of good internship training is that the hospitals are adequately staffed and equipped and ready to deliver teaching and training. Our data suggested that this is not always the case and are consistent with previous qualitative research suggesting that district hospitals provided limited learning opportunities and supervision with limited suitability as internship sites [9].

The potential consequences of poor training and supervision during internship are therefore worth consideration. As well as failing to consolidate knowledge and skills in major specialties interns may also develop burnout and stress-related psychological problems more rapidly [9]. These stresses and being forced to take significant clinical responsibility in poorly-resourced hospitals may push medical officer interns to leave the profession or the public sector which is often most resource constrained as soon as they are licensed/registered [22]. This will likely worsen the internal and external brain-drain undermining universal health coverage and equity and limiting any benefits from public investment in medical education [22]. Inadequate support and supervision also threatens

For peer review only

patient safety by creating the conditions for significant medical errors to occur. Even in settings without a ‘blame culture’ such errors can have profound effects on the health workers as well as the patients further exacerbating workers’ psychological distress [23,24]. We must also remember that todays’ medical officer interns will become tomorrows general medical officers who are often then responsible for the training of the next cohorts of interns and other health worker cadres [25]. Therefore, poor internship training for medical officers continuously compromises the quality of training and skills that are passed on and this may be a particular challenge to patient safety and healthcare quality that rely so heavily on effectively functioning teams [26].

Our analyses have policy implications for Kenya policy-makers. The regulator requires internship hospitals to maintain the minimum requirement of staff and be able to offer a core set of quality services with self-report against such requirements at least once a quarter. Our data would suggest many internship hospitals in Kenya might not continuously achieve these minimum requirements. Should this prevent interns from being licensed or should interns be reallocated to centres meeting regulatory requirements? While more stringent and regular audit and re-accreditation of internship training centres might be conducted by the regulatory council to ensure that only the hospitals that meet the minimum requirements are allowed to receive and train interns, adequate mitigation measures need to be in place so that interns themselves are not disadvantaged further. For example, by rotating interns between different level hospitals with different level of resource availability. More widely, our findings point to the need to carefully consider the potential consequences of rapidly expanding medical training and of appropriate planning and financing for new internship centres, especially in rural areas.

For peer review only

For peer review only - http://bmjopen.bmj.com/site/about/guidelines.xhtml

Several limitations should be noted for the current analysis. To start with, our analysis was only limited to the 61 internship training hospitals sampled by KHFA, mostly public hospitals. A total of 13 hospitals were not sampled in KHFA and are either private hospitals or mission hospitals, therefore comparison by hospital ownership was not feasible. Second, aside from the KHFA's own limitation on data missing, we noted data inaccuracy and inconsistency in KHFA data we retrieved. We made efforts to clean these data especially on human resources through correspondence input though we were unable to validate all the number from the KHFA survey. Third, to assess the capacity of internship training hospitals we selected 166 signal indicators from the 3000 questions from KHFA survey. Our criteria were to ensure that the indicators selected are the minimum requirement and should be achievable (i.e. should be "1" for all indicators). Our selection was guided by the national guidelines and agreed by four authors, three of whom have experience supervising interns. However, we acknowledge that this process is somewhat subjective. Fourth, we only focused on the structural and organizational features of internship training hospitals. We did not include indicators on process or outcome indicators e.g. training or patient outcomes due to limited data availability. Ideally these should be considered while evaluating internship training hospitals. Last, the KHFA data was collected in a snapshot in 2018 prior to Covid-19. The appointment and posting of specialists may fluctuate from time to time but Covid-19 specifically could have led to the government investing in hospital infrastructure, equipment and emergency hiring to improve their response capacity to Covid. Therefore it is possible that the capacity of Kenyan internship training centres has improved since 2018 [27]. Despite these limitations our data do suggest important shortcomings in internship training centres in Kenya. We suggest other LMICs that are

For peer review only - http://bmjopen.bmj.com/site/about/guidelines.xhtml

rapidly expanding their medical training should also evaluate their internship training sites to explore the generalisability of our findings.

Conclusion

We assessed the capacity of 61 Kenyan internship training hospitals using data from KHFA 2018. Our results highlighted major gaps in staffing, equipment and service availability. More specifically, there are weaknesses in areas regarding organisational arrangements that support quality and safety. Smaller hospitals are more likely to have a lower capacity index, and should be re-accredited more stringently and regularly and also be provided with adequate mitigation measures so they can provide appropriate and well-resourced training for medical interns allowing them to become fully competent medical doctors.

For peer review only

List of abbreviations

CPAP, continuous positive airway pressure

CT, computerised tomography

DHIS2, District Health Information Software-2

IPC, infection prevention and control

IQR, inter-quarter range

KHFA, Kenya Harmonized Health Facility Assessment

LMIC, low- and middle-income country

MFL, Kenya Health Master Facility List

OBGYN, obstetrics and gynaecology

PCR, polymerase chain reaction

For peer review only - http://bmjopen.bmj.com/site/about/guidelines.xhtml

Footnote

Online supplemental material 1. 166 Indicators included for each general domain and speciality domain

Online supplemental material 2. Comparing internship hospitals sampled in KHHFA and others

Online supplemental material 3. All indicator heatmap by hospital bed number

Acknowledgements: Not applicable.

Funding: YZ is supported by the University of Oxford Clarendon Fund Scholarship. ME is supported by a Wellcome Trust Senior Research Fellowship (#207522). HK is employed by the Kenya Ministry of Health.

Competing interests: The authors declare that they have no competing interests.

Patient consent for publication: Not applicable.

Authors' contributions: YZ and ME conceived of the analysis. YZ, BO, FW and ME contributed to data analysis. YZ wrote the first draft of the manuscript. BO, FW, HK, DG, CN and ME provided critical feedback on the first draft of the manuscript. All authors read and approved the final manuscript.

Data sharing statement: The data that support the findings of this study are available from Kenya Ministry of Health but restrictions apply to the availability of these data, which were used under license for the current study, and so are not publicly available. All requests for further use of these data can be made through and with the permission of the Kenya Ministry of Health.

Ethics approval: The Kenya Ministry of Health, Division of Monitoring and Evaluation, sought approval for, planned and execute the 2018 Kenya Harmonized Health Facility

For peer review only

479 Assessment. Written informed consent was sought by in-charge or acting in-charge of all the facilities surveyed. Permission was then granted to use these data for the secondary analysis reported here with the purpose of improving quality of health service provision in Kenya.

For peer review only

For peer review only - http://bmjopen.bmj.com/site/about/guidelines.xhtml

Reference

1 Ndetei DM, Mathai M, Khasakhala LI, *et al.* University medical education in Kenya: The challenges. *Medical Teacher* 2010;**32**:812–5. doi:10.3109/0142159X.2010.496011

2 MP&DC. National guidelines and log book for medical officer interns. http://medicalboard.co.ke/resources/NATIONAL%20GUIDELINES%20AND%20LOG%20BOOK%20FOR%20MEDICAL%20OFFICER%20INTERNS.pdf (accessed 5 Aug 2020).

3 Kupersmith J. Quality of care in teaching hospitals: a literature review. *Academic Medicine* 2005;**80**:458–66.

4 Colwell NP. The Requirement of an Intern Hospital. *Journal of the American Medical Association* 1929;**92**:1031–3. doi:10.1001/jama.1929.02700390001001

5 Ogero M, Akech S, Malla L, *et al.* Examining which clinicians provide admission hospital care in a high mortality setting and their adherence to guidelines: an observational study in 13 hospitals. *Arch Dis Child* 2020;**105**:648–54. doi:10.1136/archdischild-2019-317256

6 English M, Strachan B, Esamai F, *et al.* The paediatrician workforce and its role in addressing neonatal, child and adolescent healthcare in Kenya. *Archives of Disease in Childhood* 2020;**105**:927–31. doi:10.1136/archdischild-2019-318434

7 Kenya Ministry of Health. Kenya Health Workforce Report: The Status of Healthcare Professionals in Kenya, 2015. 2017. https://taskforce.org/wp-content/uploads/2019/09/KHWF_2017Report_Fullreport_042317-MR-comments.pdf (accessed 23 Jan 2020).

8 Kenya Ministry of Health. Kenya Human Resource for Health Strategic Plan 2019-2023 (Final draft). 2019.

9 Muthaura PN, Khamis T, Ahmed M, *et al.* Perceptions of the preparedness of medical graduates for internship responsibilities in district hospitals in Kenya: a qualitative study. *BMC Med Educ* 2015;**15**:178. doi:10.1186/s12909-015-0463-6

10 Approved Internship Centres- Kenya Medical Practitioners and Dentists Council. https://medicalboard.co.ke/functions/approved-internship-centres/ (accessed 12 Feb 2020).

11 Njiri F. Strengths And Challenges Of Decentralized Health Facilities As Medical Training And Research Sites In Kenya. Published Online First: June 2013.http://erepository.uonbi.ac.ke/handle/11295/61812 (accessed 11 Mar 2021).

12 Muasya MK, Dienya TM, Wagaiyu EG, *et al.* Capacity of non-tertiary Kenyan health facilities selected for decentralised dental training. *East African Medical Journal* 2016;**93**:55–9.

For peer review only

For peer review only - http://bmjopen.bmj.com/site/about/guidelines.xhtml

13 Donabedian A. The quality of care. How can it be assessed? *JAMA* 1988;**260**:1743–8. doi:10.1001/jama.260.12.1743

14 Kenya Ministry of Health. Kenya Health Facility Assessment (KHFA) 2018/19 Reports. https://www.health.go.ke/resources/downloads/ (accessed 9 Mar 2021).

15 Kenya Master Health Facility List: Find all the health facilities in Kenya. http://kmhfl.health.go.ke/#/home (accessed 9 Mar 2021).

16 Capinpuyan ALC, Miguel RTD. Ethical Challenges in Medical Community Internships. *Health Hum Rights* 2019;**21**:141–7.

17 Bola S, Trollip E, Parkinson F. The state of South African internships: A national survey against HPCSA guidelines. *S Afr Med J* 2015;**105**:535. doi:10.7196/SAMJnew.7923

18 Wilson RM, Michel P, Olsen S, *et al.* Patient safety in developing countries: retrospective estimation of scale and nature of harm to patients in hospital. *BMJ* 2012;**344**:e832. doi:10.1136/bmj.e832

19 Meyer A-C, Ndetei D. *Providing Sustainable Mental Health Care in Kenya: A Demonstration Project*. National Academies Press (US) 2016. https://www.ncbi.nlm.nih.gov/books/NBK350312/ (accessed 12 Mar 2021).

20 *Increasing Access to Health Workers in Remote and Rural Areas Through Improved Retention: Global Policy Recommendations*. Geneva: : World Health Organization 2010. http://www.ncbi.nlm.nih.gov/books/NBK138618/ (accessed 25 Feb 2020).

21 Kibore MW, Daniels JA, Child MJ, *et al.* Kenyan medical student and consultant experiences in a pilot decentralized training program at the University of Nairobi. *Educ Health (Abingdon)* 2014;**27**:170–6. doi:10.4103/1357-6283.143778

22 Essa ZI. *Post-medical interns' reflections on medical internships in South African state training hospitals.* 2011.http://wiredspace.wits.ac.za/handle/10539/9209 (accessed 11 May 2021).

23 Goddard AF, Patel M. The changing face of medical professionalism and the impact of COVID-19. *The Lancet* 2021;**397**:950–2. doi:10.1016/S0140-6736(21)00436-0

24 Wu AW, Steckelberg RC. Medical error, incident investigation and the second victim: doing better but feeling worse? *BMJ Qual Saf* 2012;**21**:267–70. doi:10.1136/bmjqs-2011-000605

25 Kenya Scheme of service for medical officers' dental officers and pharmacists. 2017. https://www.cog.go.ke/media-multimedia/reportss/category/101-schemes-of-service?download=260:scheme-of-service-for-medical-officers-dental-officers-and-pharmacists

For peer review only - http://bmjopen.bmj.com/site/about/guidelines.xhtml

26 Doctors may learn bad habits at teaching hospitals with safety violations. STAT. 2018.https://www.statnews.com/2018/04/20/doctors-learn-bad-habits-teaching-hospitals/ (accessed 3 Apr 2021).

27 Barasa EW, Ouma PO, Okiro EA. Assessing the hospital surge capacity of the Kenyan health system in the face of the COVID-19 pandemic. *PLoS One* 2020;**15**. doi:10.1371/journal.pone.0236308

For peer review only

Table 1. Domain and indicator summary

Category	Domain	Number of indicators	Example indicators	Indicator and domain conversion
Human resources	(a) Human resource for health	6	Total staff assigned, employed, seconded (including part time) – Paediatricians, Neonataologists	All the indicators were converted into binary responses with 1 representing "available" and 0 representing "unavailable". For most indicators on availability, we defined available as "onsite, observed, non-expired and functional". For tests that are reported to be available offsite, or tests that are observed but expired, and for equipment reported available but not observed, or not available now, we labeled them as "unavailable". We also considered an indicator to be unavailable if the health facility did not answer this question, usually because its filter questions were answered no.
Diagnostics and supportive care	(b) Laboratory test (including 4 sub-domains, rapid test, basic lab test, infectious diseases test, advanced lab test)	31	TB test availability - Xpert MTB/RIF rapid diagnostic testing for tuberculosis	
	(c) Oxygen and respiratory support	10	Please tell me if the pulse oximeter are available anywhere in the outpatient service area and are functional.	
	(d) General equipment	8	Electrocardiogram (ECG) Equipment available and functioning today	
Service continuity and safety	(e) 24/7 availability	8	Is emergency medicine specialist or general medical practitioners always available 24-hours for emergency service, either onsite in emergency unit or not onsite in emergency unit but on-call inside facility?	
	(f) Infectious prevention and control	9	Does this facility have guidelines or protocols for cleaning the facility such as the floors, counters, and beds?	
	(g) Quality and safety	24	How frequently does the quality assurance committee meet? - Monthly	
Major specialties	(h) Surgery (including 2 sub-domains, equipment/medicine and service)	20	Functioning of basic surgical equipment - EKG electrodes	
	(i) Internal medicine (including 2 sub-domains, equipment/medicine and service)	12	Do providers in this facility diagnose and/or manage diabetic patients?	
	(j) Obstetrics-gynaecology (including 2 sub-domains, equipment/medicine and service)	11	Parenteral administration of antibiotics (IV or IM) for mothers carried out	
	(k) Paediatrics (including 2 sub-domains, equipment/medicine and service)	20	Is KMC (Kangaroo mother care) for premature/very low birth-weight babies) used in this facility?	
	(l) Mental health	6	Does this facility offer any services for mental and/or neurological conditions?	

For peer review only - http://bmjopen.bmj.com/site/about/guidelines.xhtml

Table 2. Capacity scores by hospital characteristics

	Level 4 small hospitals (n=23)	Level 4 large hospitals (n=23)	Level 5 & 6 hospitals (n=15)	Total (n=61)
Total capacity index	63%	67%	81%	69%
Human resources for health	64%	67%	89%	71%
Lab test	60%	63%	77%	65%
Oxygen & respiratory support	64%	57%	77%	64%
General equipment	53%	59%	72%	60%
24/7	58%	65%	68%	63%
Quality and safety	35%	38%	50%	40%
Infection prevention & control	57%	58%	92%	66%
Surgery	94%	89%	97%	93%
Internal medicine	70%	73%	90%	76%
Paediatrics	67%	70%	81%	72%
OBGYN	89%	91%	91%	90%
Mental health/Neurology	44%	73%	83%	64%

Table 3. Human resources for health cadres by hospital characteristics

	Level 4 small hospitals (n=23)	Level 4 large hospitals (n=23)	Level 5 & 6 hospitals (n=15)	Total (n=61)
Medical officer	12 (6, 18)	14 (10, 20)	29 (21, 58)	14 (10, 26)
Medical specialist	2 (1, 7)	4 (1, 11)	16 (2, 26)	4 (1, 11)
Clinical officer	20 (13, 26)	25 (15, 33)	35 (21, 44)	25 (15, 33)
Nurse and midwife	206 (95, 248)	150 (113, 210)	336 (267, 534)	210 (114, 323)
Medical laboratory technologist	13 (11, 14)	14 (11, 18)	23 (19, 46)	14 (12, 20)
Radiographer	4 (3, 5)	4 (2, 6)	9 (6, 22)	5 (3, 7)
Allied health professional	11 (3, 14)	14 (10, 17)	29 (24, 44)	14 (6, 24)

Note: Data are presented as median (interquartile range), allied health professional refers to physical therapist, occupational therapist, orthopedic technician, paster technician, nutritionists and dieticians

For peer review only

Figure legends

Figure 1. Distribution of capacity score by domain

Figure 2. Number of internship training centres with different numbers of specialists (a) and with no surgeon, internists, obstetrician-gynaecologist, paediatrician and anaesthesiologist (b)

Note: We have combined different types of surgeons (general surgeons, paediatric surgeons, orthopaedic surgeons, cardiothoracic surgeon, ENT surgeons, plastic surgeons, neuro-surgeons, urological surgeons) into "surgeon" category. Similarly, specialist physicians (internists), oncologist, neurologist, cardiologist, critical care, gastroentologist, palliative care specialist, nephrologists, rheumatologist, medical endocrinologist into "internist" category, and paediatricians and neonatologists into "paediatrician" category.

Figure 3. Average lab test score by sub-domain and hospital characteristics

For peer review only

For peer review only - http://bmjopen.bmj.com/site/about/guidelines.xhtml

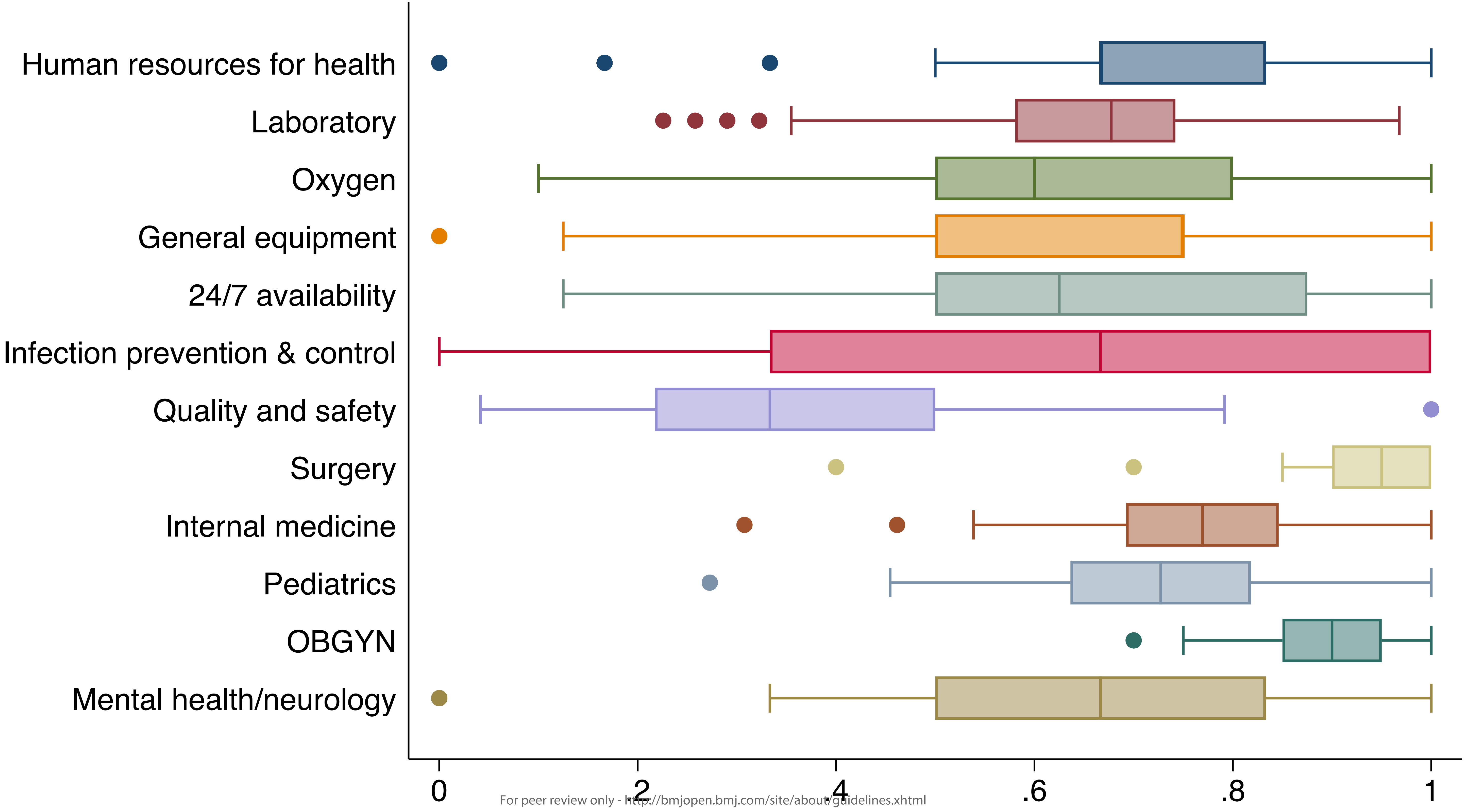
Human resources for health
Laboratory
Oxygen
General equipment
24/7 availability
Infection prevention & control
Quality and safety
Surgery
Internal medicine
Pediatrics
OBGYN
Mental health/neurology
0
.2
.4
.6
.8
1

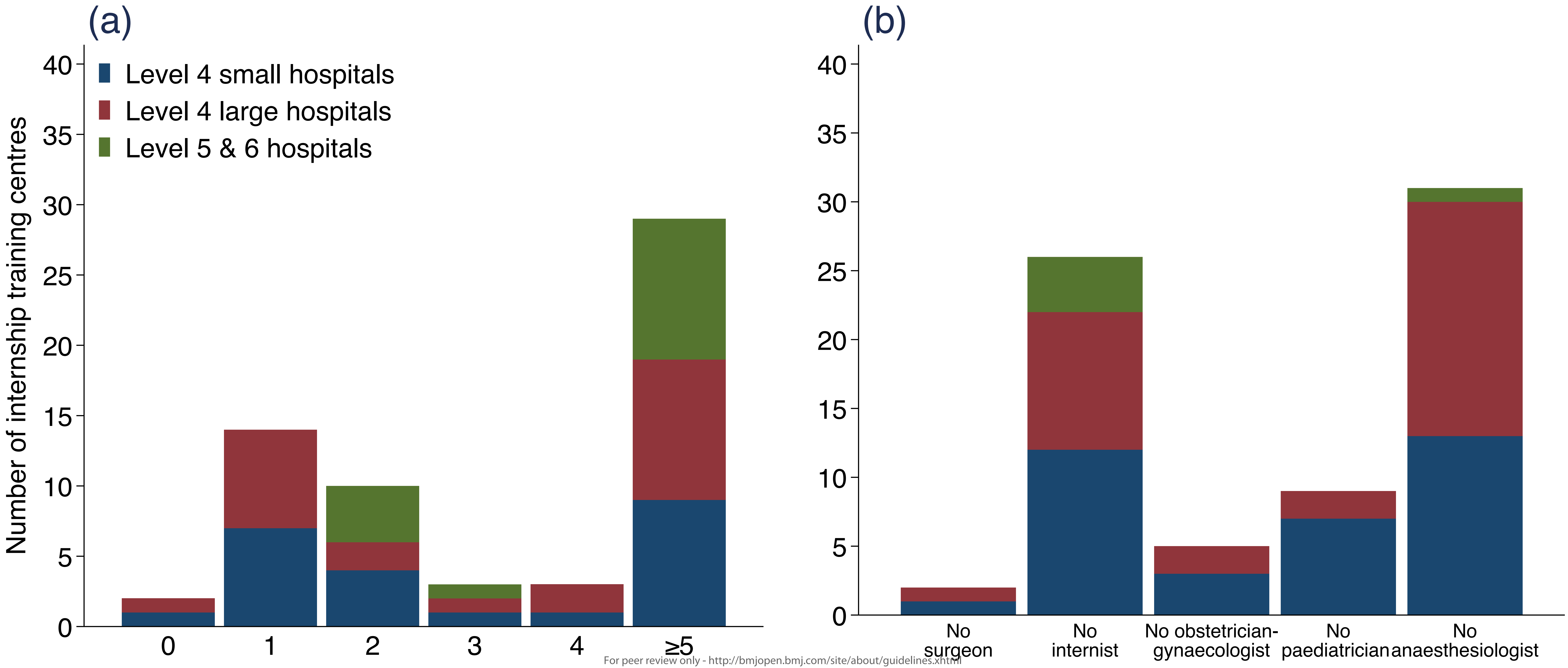
(a)
Number of internship training centres
40
35
30
25
20
15
10
5
0
Level 4 small hospitals
Level 4 large hospitals
Level 5 & 6 hospitals
0
1
2
3
4
≥5
(b)
40
35
30
25
20
15
10
5
0
No surgeon
No internist
No obstetrician-gynaecologist
No paediatrician
No anaesthesiologist

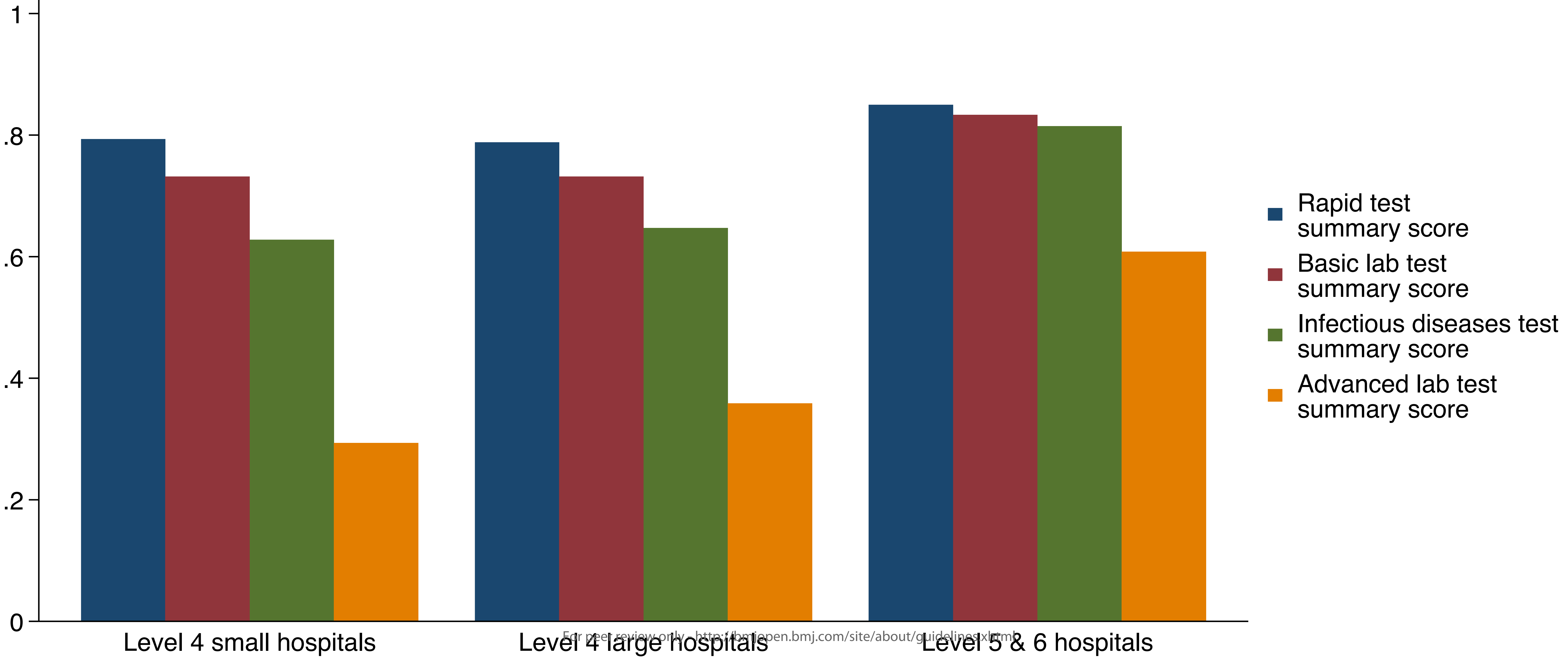
1
.8
.6
.4
.2
0
Level 4 small hospitals
Level 4 large hospitals
Level 5 & 6 hospitals
Rapid test summary score
Basic lab test summary score
Infectious diseases test summary score
Advanced lab test summary score

Additional file 1. 166 Indicators included for each general domain and speciality domain

Domain	Indicator	1 – considered available	0 – considered unavailable
**Human resources for health (6)**	At least five medical specialists	- 5 or more specialists	- Less than 5 specialists
	At least one surgeon	- 1 or more	- Less than 1
	At least one internist	- 1 or more	- Less than 1
	At least one paediatrician	- 1 or more	- Less than 1
	At least one obstetrician-gynaecologist	- 1 or more	- Less than 1
	At least one anaesthesiologist	- 1 or more	- Less than 1
**Lab test (31)**	HIV rapid test	- Observed, at least one not expired	- Not observed, reported available but not seen<br>- Not observed, never available
	Syphilis rapid test	- Observed, at least one not expired	- Not observed, reported available but not seen<br>- Not observed, never available
	Urine rapid test for pregnancy	- Observed, at least one not expired	- Not observed, reported available but not seen<br>- Not observed, never available
	Urine dipstick 3	- Observed, at least one not expired	- Not observed, reported available but not seen<br>- Not observed, never available
	Haemoglobin A1c rapid test	- Observed, at least one not expired	- Not observed, reported available but not seen<br>- Not observed, never available
	Colorimeter or haemoglobinometer	- Observed, functional/valid	- Reported (not seen), functional/valid<br>- Not available today
	Glucometer	- Observed, functional/valid	- Reported (not seen), functional/valid<br>- Not available today
	Glucometer test strips/discs	- Observed, functional/valid	- Reported (not seen), functional/valid<br>- Not available today
	Any blood chemistry test	- Yes, onsite	- No, never available
	Renal function test	- Yes, observed, functional/valid	- No, not available today
	Other tests for bilirubin	- Yes, onsite	- Yes, offsite (specimen sent)<br>- No, never available
	ABO blood grouping testing	- Yes, onsite	- Yes, offsite (specimen sent)<br>- No, never available
	Rhesus blood grouping testing	- Yes, onsite	- Yes, offsite (specimen sent)<br>- No, never available
	Cross-match testing	- Yes, onsite	- Yes, offsite (specimen sent)<br>- No, not available today
	Onsite PCR for HIV viral load or HIV early-infant diagnosis	- Yes, onsite	- Yes, offsite (specimen sent)<br>- Yes, not fucntional/not valid<br>- No, not available today
	Xpert MTB/RIF rapid diagnostic testing for TB	- Yes, onsite	- Yes, offsite (specimen sent)<br>- No, not available today
	Urine microscopy	- Yes, observed, functional/valid	- No, not available today
	Malaria smears	- Yes, observed, functional/valid	- No, not available today
	Microscopy test CSF body fluid counts	- Yes, onsite	- Yes, offsite (specimen sent)<br>- No, never available
	Cryptococcal antigen testing	- Yes, onsite	- Yes, offsite (specimen sent)<br>- No, never available
	Gram stain testing	- Yes, onsite	- Yes, offsite (specimen sent)<br>- No, never available
	Culture and sensitivity test	- Yes, onsite	- Yes, offsite (specimen sent)<br>- No, never available
	Blood cultures test	- Yes, onsite	- Yes, offsite (specimen sent)<br>- No, never available
	Fine needle aspiration cytology	- Perform in facility	- Refer for test<br>- Not used
	Core needle biopsy of lump specimen	- Perform in facility	- Refer for test<br>- Not used
	Prostate Specific Antigen (PSA) Testing	- Perform in facility	- Refer for test<br>- Not used
	Blood gas measurement	- Yes, onsite	- Yes, offsite (specimen sent)

For peer review only

			- No, never available
	Cardiac marker (CK, Troponin) test	- Yes, onsite	- Yes, offsite (specimen sent)<br>- No, never available
	PAP smear read in facility	- Yes, read in facility	- Yes, read outside facility<br>- No<br>- 0 PAP smears were conducted with results recorded during the past 12 completed months
	VIA/VILLI read in facility	- Yes, read in facility	- Yes, read outside facility<br>- No<br>- 0 VIA/VILLI were conducted with results recorded during the past 12 completed months
	Prepare and examine tissues or samples for cancer	- Yes	- No
**Oxygen and respiratory support (10)**	Surgery oxygen equipment functioning - Oxygen tank/cylinder with attached pressure gauge, pressure regulator	- Yes	- No<br>- Don't know
	Maternity paediatric-sized oxygen delivery apparatus functioning	- Yes	- No
	Outpatient pulse oximeter functioning	- Yes	- No
	Maternity pulse oximeter functioning	- Yes	- No
	Paediatrics/neonatal oxygen functioning	- Yes	- No
	Emergency pulse oximeter functioning	- Yes	- No
	Ventilator available	- Yes, available now	- No
	Chest tubes available	- Observed, at least one not expired	- Not observed, not available today<br>- Not observed, never available
	Adult CPAP equipment available	- Observed, at least one not expired	- Observed, available but expired<br>- Not observed, reported available but not seen<br>- Not observed, not available today<br>- Not observed, never available
	Emergency paediatrics and neonatal CPAP available	- Observed	- Reported, not seen<br>- Not available
**General equipment (8)**	ECG offered	- Yes	- No
	ECG equipment functioning	- Yes equipment available and functioning	- No equipment not available or not functioning
	Ultrasound offered	- Yes	- No
	Ultrasound equipment functioning	- Yes equipment available and functioning	- No equipment not available or not functioning
	X-ray offered	- Yes	- No
	X-ray equipment functioning	- Yes equipment available and functioning	- No equipment not available or not functioning
	CT scan offered	- Yes	- No
	CT scan equipment functioning	- Yes equipment available and functioning	- No equipment not available or not functioning
**24 7 (8)**	Emergency service with structured triage tool 24/7	- 24-hour emergency service	- 10 hours<br>- No structured triage tool
	Medical officer onsite or oncall in facility 24/7	- MO or Emergency practitioner always available, onsite<br>- MO or Emergency practitioner always available, not onsite but oncall in facility	- MO or Emergency practitioner always available, not in facility<br>- Staff not available 24 hours
	Formal triage tool used 24/7	- Yes	- No
	Emergency surgery and anaesthesia 24/7	- 24-hour emergency service	- 0, 2 or 8 hours
	Emergency radiology 24/7	- 24-hour emergency service	- 0, 5, 9, 10, 12 hours
	Emergency diagnostics 24/7	- 24-hour emergency service	- 0, 7, 10, 12 hours
	Emergency pharmacy 24/7	- 24-hour emergency service	- 8, 9, 10, 12, 15 hours<br>- No dedicated pharmacy in facility
	Blood transfusion	- Yes available now	- No
**IPC (9)**	Facility has IPC guideline	- Yes, observed	- Reported not seen<br>- No
	Technical IPC committee	- Yes	- No
	Dedicated (full-time) IPC staff	- Yes	- No

For peer review only

	Guidelines for cleaning	- Yes, observed	- Reported not seen<br>- No
	Step-by-step techniques for specific tasks	- Yes	- No
	Cleaning roster or schedule specifying responsibility	- Yes	- No
	Cleaning beds observed	- Yes	- No
	Cleaning counters/tables observed	- Yes	- No
	Cleaning toilets observed	- Yes	- No
**Quality and safety (24)**	Monthly quality assurance committee meeting	- Monthly	- Quarterly<br>- Biannually
	Documentation of quality assurance information reviewed	- Yes, documentation observed	- Yes, documentation reported not seen
	Ongoing quality improvement using Plan-Do-Check-Study-Act approach	- Yes, service-specific<br>- Yes, across facility	- No
	Multi-disciplinary quality improvement team	- Yes	- No
	Departmental specific work improvement teams	- Yes	- No
	Documentation of improvement achieved as a result of quality improvement process	- Yes, documentation observed	- Yes, documentation reported not seen<br>- No
	Kenya Quality Model for Health completed in 2017 or 2018	- 2017<br>- 2018	- 2014<br>- 2016<br>- Don't know
	Formal case reviews carried out at least monthly	- At least weekly<br>- At least monthly	- At least quarterly<br>- No specific timing
	Death reviews results recorded	- Yes	- No
	Guidelines of events that are considered adverse and required to be reported	- Yes, documentation observed	- Yes, documentation reported not seen<br>- No
	Notes or reports that show evidence of review and plan of action for the reports about adverse events	- Yes, documentation observed	- Yes, documentation reported not seen<br>- No
	Written guidelines that define nosocomial infections and the process for reporting	- Yes, observed	- Reported not seen<br>- No
	Report or record shows nosocomial infections reported over the past 6 months	- Yes, observed	- Reported not seen<br>- No
	Written policies and procedures for identifying and managing drug-use problems	- Yes, observed	- Reported not seen<br>- No
	Surgery related mortality and morbidity review meetings at least monthly	- At least weekly<br>- At least monthly	- At least quarterly<br>- No specific timing<br>- None conducted
	Surgery related mortality and morbidity review meeting results recorded	- Yes, observed	- Reported not seen<br>- No
	Written guidelines or instructions for reporting on adverse events related to surgery	- Yes, observed	- Reported not seen<br>- No<br>- Don't know
	Notes or reports that show evidence of review and plan of action for the surgery reports	- Yes, documentation observed	- Yes, documentation reported not seen<br>- No
	Guidelines of postoperative infection definition	- Yes, documentation observed	- Yes, documentation reported not seen<br>- No
	Notes or reports that show evidence of review and plan of action for the reports about postoperative infection	- Yes, documentation observed	- Yes, documentation reported not seen<br>- No
	Indicator on deaths prior to discharge among patients who had a procedure in a surgical theater monitored	- Yes	- No
	Indicator on deaths prior to discharge among < 15 year old patients who had a procedure in a surgical theater monitored	- Yes	- No
	Indicator on post-operative surgical wound infection numbers monitored	- Yes	- No
	Facility monitor unplanned and unexpected hospital readmissions for any conditions	- Yes	- No
**Surgery equipment and medication (8)**	Basic operating table functioning	- Yes	- No
	Overhead operating light functioning	- Yes	- No
	Cardiac monitor functioning	- Yes	- No

For peer review only - http://bmjopen.bmj.com/site/about/guidelines.xhtml

			- Don't know
	EKG electrodes functioning	- Yes	- No<br>- Don't know
	Thermometer functioning	- Yes	- No<br>- Don't know
	Blood pressure apparatus functioning	- Yes	- No
	Suction apparatus functioning	- Yes	- No
	Anaesthesia machine available	- Yes available now	- No
**Surgery service (12)**	Wound debridement service available	- Yes, outpatient<br>- Yes, inpatient<br>- Yes both out and inpatient	- No
	Acute burn management service available	- Yes, outpatient<br>- Yes, inpatient<br>- Yes both out and inpatient	- No
	Closed repair of fracture service available	- Yes, outpatient<br>- Yes, inpatient<br>- Yes both out and inpatient	- No
	Closed reduction of dislocated joint service available	- Yes, outpatient<br>- Yes, inpatient<br>- Yes both out and inpatient	- No
	Cricothyroidotomy service available	- Yes, outpatient<br>- Yes, inpatient<br>- Yes both out and inpatient	- No
	Male circumcision service available	- Yes, outpatient<br>- Yes, inpatient<br>- Yes both out and inpatient	- No
	Hydrocele reduction service available	- Yes, outpatient<br>- Yes, inpatient<br>- Yes both out and inpatient	- No
	Biopsy of lymph node or mass service available	- Yes, outpatient<br>- Yes, inpatient<br>- Yes both out and inpatient	- No
	Appendectomy service available	- Yes, outpatient<br>- Yes, inpatient<br>- Yes both out and inpatient	- No
	Hernia repair service available	- Yes, outpatient<br>- Yes, inpatient<br>- Yes both out and inpatient	- No
	Open reduction and fixation service available	- Yes, outpatient<br>- Yes, inpatient<br>- Yes both out and inpatient	- No
	Any procedures using laparotomy service available	- Yes, outpatient<br>- Yes, inpatient<br>- Yes both out and inpatient	- No
**Internal medicine equipment and medication (3)**	Defibrillator available	- Yes, available now	- No
	Renal dialysis/haemodialysis machine available	- Yes, available now	- Yes, not available now<br>- No
	Lumbar puncture kit available	- Observed, at least one not expired	- Observed, available but expired<br>- Not observed, reported available but not seen<br>- Not observed, not available today<br>- Not observed, never available
**Internal medicine service (10)**	ART prescription and follow-up for U5 children service available	- Yes, ART prescription and clinical follow-up	- Yes, ART prescription, no clinical follow-up<br>- Yes, clinical follow-up, no ART prescription
	HIV/AIDS care and support service available	- Yes	- No
	Diagnose and/or manage diabetes available	- Yes	- No
	Diagnose and/or manage acute myocardial infraction available	- Diagnose, treat and patient follow-up	- Patient follow-up only<br>- No service/refer suspect case
	Diagnose and/or manage chronic respiratory disease available	- Yes	- No
	Diagnose and/or manage chronic kidney disease available	- Yes	- No
	Screening, diagnosis and/or treatment services for breast cancer available	- Yes	- No

For peer review only

	Screening, diagnosis and/or treatment services for colorectal cancer available	- Yes	- No
	Screening, diagnosis and/or treatment services for prostate cancer available	- Yes	- No
	Palliative care service available	- Yes	- No
**Paediatrics equipment and medication (8)**	Phototherapy machine available	- Yes, available now	- No
	Infant incubator available at outpatient	- Yes, available now	- Yes, not available now<br>- No
	Bed or location for KMC caregiver overnight	- Yes, observed	- Reported, not seen<br>- No
	Exchange transfusion blood available today	- Yes	- No
	Incubator available today	- Yes	- No
	Radiant warmer available today	- Yes	- No
	Artificial ventilation available today	- Yes	- No
	Device for intraosseous access available	- Observed, at least one not expired	- Observed, available but expired<br>- Not observed, reported available but not seen<br>- Not observed, not available today<br>- Not observed, never available
**Paediatrics service (3)**	KMC for premature or LBW used	- Yes	- No
	Newborn sepsis service available	- Yes	- No
	Newborn sepsis full antibiotics regimen and follow-up always available	- Yes always	- Yes sometimes<br>- Never
**OBGYN equipment and medication (10)**	Blood pressure apparatus functioning	- Yes	- No
	Infant scale (with 100 gram gradation) functioning	- Yes	- Don't know
	Resuscitation table (with heat source) functioning	- Yes	- No
	Self-inflating bag and mask for resuscitation functioning	- Yes	- No
	Newborn bag and mask size 0 for resuscitation of pre-term babies functioning	- Yes	- No
	Newborn bag and mask size 1 for resuscitation of term babies functioning	- Yes	- No
	Magnesium sulphate injection available	- Observed, at least one not expired	- Not observed, not available today
	Dexamethasone injection available	- Observed, at least one not expired	- Not observed, not available today<br>- Not observed, never available
	Misoprostol tablet 200 mcg available	- Observed, at least one not expired	- Not observed, not available today<br>- Not observed, never available
	Oxytocin injection available	- Observed, at least one not expired	- Not observed, not available today<br>- Not observed, never available
**OBGYN service (10)**	Active management of third stage labour (AMTSL) service available	- Yes	- No
	Monitoring and management of labor using a partograph service available	- Yes	- No
	Hygienic cord care service available	- Yes	- No
	Parenteral administration of antibiotics carried out	- Yes	- No
	Parenteral administration of oxytocic for treatment of postpartum haemorrhage carried out	- Yes	- No
	Assisted vaginal delivery using manual vacuum extraction (MVE) or forceps carried out	- Yes	- No, service not offered<br>- No, trained staff but no cases
	Manual removal of placenta carried out	- Yes	- No
	Removal of retained products of conception using D&C or manual vacuum aspiration carried out	- Yes	- No, service not offered
	PMTCT available	- Yes	- No
	Treatment of pre-invasive cervical cancer available	- Yes	- No
**Mental health / neurology (6)**	Any mental/neurological services offered	- Yes, inpatient only	- Not offered
	Mental health inpatient ward available	- Yes, inpatient only	- Not offered

For peer review only

	Neurological health inpatient ward available	- Yes, inpatient only	- Not offered
	Depression diagnosis and follow-up available	- Diagnoses and provides patient follow-up	- Diagnoses only<br>- Provides patient follow-up only<br>- No
	Psychosis diagnosis and follow-up available	- Diagnoses and provides patient follow-up	- Diagnoses only<br>- Provides patient follow-up only<br>- No
	Epilepsy diagnosis and follow-up available	- Diagnoses and provides patient follow-up	- Diagnoses only<br>- Provides patient follow-up only<br>- No

For peer review only

For peer review only

Additional file 2. Comparing internship hospitals sampled in KHHFA and others

	61 KHHFA hospitals	12 other hospitals
Level		
- Level 4	44	11
- Level 5	15	1
- Level 6	2	0
Ownership		
- MoH	54	0
- Private	2	4
- FBO	5	8
Bed number		
- Mean (SD)	262.9 (213.4)	171.2 (71.0)
- Median (IQR)	200 (156, 276)	180 (106, 221)
Total delivery		
- Mean	4585.1 (2747.0)	1349.4 (945.7)
- Median (IQR)	3980.7 (2972.3, 5502.7)	1150.4 (649.7, 1683.5)
Live births		
- Mean	4466.9 (2691.9)	1321.3 (927.8)
- Median (IQR)	3814.3 (2844, 5384.3)	1138.4 (626.5, 1630)
C-section		
- Mean	1190.3 (917.6)	536.1 (270.7)
- Median (IQR)	987.3 (734.3, 1319.3)	536.6 (289, 708.5)

For peer review only

Additional file 3. All indicator heatmap by hospital bed number

	Level 4 small hospitals (n=23)	Level 4 large hospitals (n=23)	Level 5 & 6 hospitals (n=15)	Total (n=61)
**Human resources for health**				
At least five medical specialists	39%	44%	67%	48%
At least one surgeon	96%	96%	100%	97%
At least one internist	48%	57%	73%	57%
At least one paediatrician	70%	91%	100%	85%
At least one obstetrician-gynaecologist	87%	91%	100%	92%
At least one anaesthesiologist	44%	26%	93%	49%
**Lab tests**				
Rapid test				
HIV rapid test	87%	91%	87%	89%
Syphilis rapid test	96%	91%	100%	95%
Urine rapid test for pregnancy	100%	91%	100%	97%
Urine dipstick 3	35%	44%	53%	43%
Haemoglobin A1c rapid test	57%	57%	87%	64%
Colorimeter or haemoglobinometer	70%	61%	67%	66%
Glucometer	96%	96%	93%	95%
Glucometer test strips/discs	96%	100%	93%	97%
Basic lab test				
Any blood chemistry test	83%	78%	93%	84%
Renal function test	70%	74%	93%	77%
Other tests for bilirubin	65%	70%	67%	67%
ABO blood grouping testing	83%	74%	80%	79%
Rhesus blood grouping testing	83%	74%	87%	80%
Cross-match testing	57%	70%	80%	67%
Infectious disease test				
Onsite PCR for HIV viral load or HIV early-infant diagnosis	0%	4%	27%	8%
Xpert MTB/RIF rapid diagnostic testing for TB	61%	83%	87%	75%
Urine microscopy	91%	83%	93%	89%
Malaria smears	91%	83%	93%	89%
Microscopy test CSF body fluid counts	83%	78%	93%	84%
Cryptococcal antigen testing	74%	78%	80%	77%
Gram stain testing	91%	78%	93%	87%
Culture and sensitivity test	57%	74%	87%	71%
Blood cultures test	17%	22%	80%	34%
Advanced lab test				
Fine needle aspiration cytology	22%	44%	67%	41%
Core needle biopsy of lump specimen	35%	44%	67%	46%
Prostate Specific Antigen (PSA) Testing	48%	61%	80%	61%
Blood gas measurement	9%	13%	60%	23%
Cardiac marker (CK, Troponin) test	9%	30%	53%	28%
PAP smear read in facility	17%	13%	33%	20%
VIA/VILLI read in facility	96%	78%	73%	84%
Prepare and examine tissues or samples for cancer	0%	4%	53%	15%

For peer review only - http://bmjopen.bmj.com/site/about/guidelines.xhtml

**Oxygen and respiratory support**				
Surgery oxygen equipment functioning - Oxygen tank/cylinder with attached pressure gauge, pressure regulator	87%	83%	73%	82%
Maternity paediatric-sized oxygen delivery apparatus functioning	87%	74%	93%	84%
Outpatient pulse oximeter functioning	39%	48%	60%	48%
Maternity pulse oximeter functioning	35%	52%	67%	49%
Paediatrics/neonatal oxygen functioning	100%	100%	100%	100%
Emergency pulse oximeter functioning	65%	65%	100%	74%
Ventilator available	52%	52%	87%	61%
Chest tubes available	30%	61%	80%	54%
Adult CPAP equipment available	13%	30%	40%	26%
Emergency paediatrics and neonatal CPAP available	17%	26%	20%	21%
**Equipment**				
ECG offered	39%	35%	93%	51%
ECG equipment functioning	26%	35%	87%	44%
Ultrasound offered	83%	91%	100%	90%
Ultrasound equipment functioning	83%	91%	100%	90%
X-ray offered	87%	87%	100%	90%
X-ray equipment functioning	87%	83%	100%	89%
CT scan offered	26%	22%	80%	38%
CT scan equipment functioning	26%	17%	73%	34%
**24 7 availability**				
Emergency service 24/7	26%	13%	47%	26%
Medical officer onsite or oncall in facility 24/7	78%	70%	93%	79%
Formal triage tool used 24/7	26%	17%	47%	28%
Emergency surgery and anaesthesia 24/7	17%	13%	40%	21%
Emergency radiology 24/7	91%	87%	100%	92%
Emergency diagnostics 24/7	96%	87%	93%	92%
Emergency pharmacy 24/7	87%	83%	93%	87%
Blood transfusion	91%	83%	100%	90%
**Infection prevention and control**				
Facility has IPC guideline	87%	70%	87%	80%
Technical IPC committee	96%	100%	93%	97%
Dedicated (full-time) IPC staff	57%	52%	73%	59%
Guidelines for cleaning	48%	57%	33%	48%
Step-by-step techniques for specific tasks	44%	57%	60%	53%
Cleaning roster or schedule specifying responsibility	48%	61%	73%	59%
Cleaning beds observed	44%	61%	67%	56%
Cleaning counters/tables observed	48%	61%	60%	56%
Cleaning toilets observed	48%	70%	67%	61%
**Quality and safety**				
Monthly quality assurance committee meeting	26%	35%	53%	36%
Documentation of quality assurance information reviewed	52%	44%	67%	53%
Ongoing quality improvement using Plan-Do-Check-Study-Act approach	64%	67%	67%	66%
Multi-disciplinary quality improvement team	65%	61%	80%	67%
Departmental specific work improvement teams	65%	61%	80%	67%
Documentation of improvement achieved as a result of quality improvement process	48%	52%	33%	46%

Kenya Quality Model for Health completed in 2017 or 2018	48%	44%	60%	49%
Formal case reviews carried out at least monthly	57%	57%	67%	59%
Death reviews results recorded	91%	91%	100%	93%
Guidelines of events that are considered adverse and required to be reported	22%	35%	33%	30%
Notes or reports that show evidence of review and plan of action for the reports about adverse events	22%	39%	27%	30%
Written guidelines that define nosocomial infections and the process for reporting	9%	9%	40%	16%
Report or record shows nosocomial infections reported over the past 6 months	9%	4%	33%	13%
Written policies and procedures for identifying and managing drug-use problems	70%	65%	67%	67%
Surgery related mortality and morbidity review meetings at least monthly	44%	39%	60%	46%
Surgery related mortality and morbidity review meeting results recorded	44%	57%	53%	51%
Written guidelines or instructions for reporting on adverse events related to surgery	9%	26%	33%	21%
Notes or reports that show evidence of review and plan of action for the surgery reports	9%	17%	27%	16%
Guidelines of postoperative infection definition	17%	26%	33%	25%
Notes or reports that show evidence of review and plan of action for the reports about postoperative infection	13%	17%	13%	15%
Indicator on deaths prior to discharge among patients who had a procedure in a surgical theater monitored	9%	22%	53%	25%
Indicator on deaths prior to discharge among < 15 year old patients who had a procedure in a surgical theater monitored	9%	17%	47%	21%
Indicator on post-operative surgical wound infection numbers monitored	9%	17%	40%	20%
Facility monitor unplanned and unexpected hospital readmissions for any conditions	30%	26%	40%	31%
**Surgery**				
Surgery equipment & medication				
Basic operating table functioning	96%	96%	100%	97%
Overhead operating light functioning	96%	96%	100%	97%
Cardiac monitor functioning	87%	83%	100%	89%
EKG electrodes functioning	83%	70%	100%	82%
Thermometer functioning	96%	87%	93%	92%
Blood pressure apparatus functioning	70%	91%	93%	84%
Suction apparatus functioning	100%	96%	100%	98%
Anaesthesia machine available	83%	74%	93%	82%
Surgery service				
Wound debridement service available	100%	100%	100%	100%
Acute burn management service available	96%	96%	100%	97%
Closed repair of fracture service available	96%	91%	100%	95%
Closed reduction of dislocated joint service available	96%	91%	100%	95%
Cricothyroidotomy service available	78%	57%	80%	71%
Male circumcision service available	100%	96%	93%	97%
Hydrocele reduction service available	100%	91%	93%	95%
Biopsy of lymph node or mass service available	100%	100%	100%	100%
Appendectomy service available	100%	96%	100%	98%
Hernia repair service available	100%	96%	100%	98%
Open reduction and fixation service available	100%	83%	100%	93%
Any procedures using laparotomy service available	100%	96%	100%	98%
**Internal medicine**				

Internal medicine equipment & medication				
Defibrillator available	57%	39%	73%	54%
Renal dialysis/haemodialysis machine available	44%	65%	93%	64%
Lumbar puncture kit available	22%	26%	80%	38%
Internal medicine service				
ART prescription and follow-up for U5 children service available	96%	96%	93%	95%
HIV/AIDS care and support service available	100%	96%	100%	98%
Diagnose and/or manage diabetes available	100%	100%	100%	100%
Diagnose and/or manage acute myocardial infraction available	83%	74%	93%	82%
Diagnose and/or manage chronic respiratory disease available	100%	100%	100%	100%
Diagnose and/or manage chronic kidney disease available	83%	91%	100%	90%
Screening, diagnosis and/or treatment services for breast cancer available	78%	87%	93%	85%
Screening, diagnosis and/or treatment services for colorectal cancer available	35%	30%	60%	39%
Screening, diagnosis and/or treatment services for prostate cancer available	61%	83%	87%	75%
Palliative care service available	48%	65%	100%	67%
**Paediatrics**				
Paediatrics equipment & medication				
Phototherapy machine available	52%	52%	67%	56%
Infant incubator available	74%	78%	87%	79%
Bed or location for KMC caregiver overnight	61%	70%	80%	69%
Exchange transfusion blood available today	26%	17%	60%	31%
Incubator available today	96%	100%	100%	98%
Radiant warmer available today	91%	96%	93%	93%
Artificial ventilation available today	48%	57%	73%	57%
Device for intraosseous access available	17%	30%	53%	31%
Paediatrics service				
KMC for premature or LBW used	83%	87%	93%	87%
Newborn sepsis service available	100%	96%	100%	98%
Newborn sepsis full antibiotics regimen and follow-up always available	91%	87%	87%	89%
**OBGYN**				
OBGYN equipment & medication				
Blood pressure apparatus functioning	91%	100%	100%	97%
Infant scale (with 100 gram gradation) functioning	87%	96%	100%	93%
Resuscitation table (with heat source) functioning	91%	100%	93%	95%
Self-inflating bag and mask for resuscitation functioning	87%	91%	80%	87%
Newborn bag and mask size 0 for resuscitation of pre-term babies functioning	91%	91%	87%	90%
Newborn bag and mask size 1 for resuscitation of term babies functioning	96%	96%	93%	95%
Magnesium sulphate injection available	96%	96%	100%	97%
Dexamethasone injection available	91%	91%	73%	87%
Misoprostol tablet 200 mcg available	74%	70%	73%	72%
Oxytocin injection available	100%	96%	100%	98%
OBGYN service				
Active management of third stage labour (AMTSL) service available	100%	100%	100%	100%
Monitoring and management of labor using a partograph service available	96%	96%	100%	97%
Hygienic cord care service available	100%	91%	93%	95%

For peer review only

Parenteral administration of antibiotics carried out	100%	100%	100%	100%
Parenteral administration of oxytocic for treatment of postpartum haemorrhage carried out	100%	100%	100%	100%
Assisted vaginal delivery using manual vacuum extraction (MVE) or forceps carried out	57%	74%	73%	67%
Manual removal of placenta carried out	100%	100%	100%	100%
Removal of retained products of conception using D&C or manual vacuum aspiration carried out	83%	83%	100%	87%
PMTCT available	100%	100%	100%	100%
Treatment of pre-invasive cervical cancer available	44%	48%	53%	48%
**Mental health / neurology**				
Any mental/neurological services offered	65%	96%	93%	84%
Mental health inpatient ward available	26%	48%	80%	48%
Neurological health inpatient ward available	13%	22%	67%	30%
Depression diagnosis and follow-up available	48%	87%	87%	72%
Psychosis diagnosis and follow-up available	52%	91%	87%	75%
Epilepsy diagnosis and follow-up available	57%	91%	87%	77%

For peer review only

STROBE Statement—Checklist of items that should be included in reports of ***cross-sectional studies***

	Item No	Recommendation	Page No
**Title and abstract**	1	(*a*) Indicate the study's design with a commonly used term in the title or the abstract	1
		(*b*) Provide in the abstract an informative and balanced summary of what was done and what was found	3-4
**Introduction**			
Background/rationale	2	Explain the scientific background and rationale for the investigation being reported	6-8
Objectives	3	State specific objectives, including any prespecified hypotheses	7-8
**Methods**			
Study design	4	Present key elements of study design early in the paper	8
Setting	5	Describe the setting, locations, and relevant dates, including periods of recruitment, exposure, follow-up, and data collection	8
Participants	6	(*a*) Give the eligibility criteria, and the sources and methods of selection of participants	8
Variables	7	Clearly define all outcomes, exposures, predictors, potential confounders, and effect modifiers. Give diagnostic criteria, if applicable	8-9
Data sources/ measurement	8*	For each variable of interest, give sources of data and details of methods of assessment (measurement). Describe comparability of assessment methods if there is more than one group	8-9
Bias	9	Describe any efforts to address potential sources of bias	8-9
Study size	10	Explain how the study size was arrived at	8-9
Quantitative variables	11	Explain how quantitative variables were handled in the analyses. If applicable, describe which groupings were chosen and why	10
Statistical methods	12	(*a*) Describe all statistical methods, including those used to control for confounding	10
		(*b*) Describe any methods used to examine subgroups and interactions	10
		(*c*) Explain how missing data were addressed	9
		(*d*) If applicable, describe analytical methods taking account of sampling strategy	N/A
		(*e*) Describe any sensitivity analyses	N/A
**Results**			
Participants	13*	(a) Report numbers of individuals at each stage of study—eg numbers potentially eligible, examined for eligibility, confirmed eligible, included in the study, completing follow-up, and analysed	10-11
		(b) Give reasons for non-participation at each stage	N/A
		(c) Consider use of a flow diagram	N/A
Descriptive data	14*	(a) Give characteristics of study participants (eg demographic, clinical, social) and information on exposures and potential confounders	11
		(b) Indicate number of participants with missing data for each variable of interest	N/A
Outcome data	15*	Report numbers of outcome events or summary measures	11-15

For peer review only - http://bmjopen.bmj.com/site/about/guidelines.xhtml

Main results	16	(*a*) Give unadjusted estimates and, if applicable, confounder-adjusted estimates and their precision (eg, 95% confidence interval). Make clear which confounders were adjusted for and why they were included	11-15
		(*b*) Report category boundaries when continuous variables were categorized	N/A
		(*c*) If relevant, consider translating estimates of relative risk into absolute risk for a meaningful time period	N/A
Other analyses	17	Report other analyses done—eg analyses of subgroups and interactions, and sensitivity analyses	11-15
**Discussion**			
Key results	18	Summarise key results with reference to study objectives	15
Limitations	19	Discuss limitations of the study, taking into account sources of potential bias or imprecision. Discuss both direction and magnitude of any potential bias	19-20
Interpretation	20	Give a cautious overall interpretation of results considering objectives, limitations, multiplicity of analyses, results from similar studies, and other relevant evidence	15-19
Generalisability	21	Discuss the generalisability (external validity) of the study results	19
**Other information**			
Funding	22	Give the source of funding and the role of the funders for the present study and, if applicable, for the original study on which the present article is based	21

*Give information separately for exposed and unexposed groups.

Note: An Explanation and Elaboration article discusses each checklist item and gives methodological background and published examples of transparent reporting. The STROBE checklist is best used in conjunction with this article (freely available on the Web sites of PLoS Medicine at http://www.plosmedicine.org/, Annals of Internal Medicine at http://www.annals.org/, and Epidemiology at http://www.epidem.com/). Information on the STROBE Initiative is available at www.strobe-statement.org.

For peer review only

For peer review only - http://bmjopen.bmj.com/site/about/guidelines.xhtml
